# Production of (R)-citramalate by engineered *Saccharomyces cerevisiae*

**DOI:** 10.1016/j.mec.2024.e00247

**Published:** 2024-08-10

**Authors:** Ryosuke Mitsui, Akihiko Kondo, Tomokazu Shirai

**Affiliations:** a Center for Sustainable Resource Science, RIKEN, 1-7-22, Suehiro-cho, Tsurumi-ku, Yokohama, Kanagawa, 230-0045, Japan; b Graduate School of Science, Technology and Innovation, Kobe University, 1-1 Rokkodai, Nada, Kobe, 657-8501, Japan

**Keywords:** *Saccharomyces cerevisiae*, (*R*)-Citramalate, Cytosolic acetyl-CoA, Phosphoketolase, Phosphotransacetylase, Dicarboxylate transporter

## Abstract

The budding yeast, *Saccharomyces cerevisiae*, has a high tolerance to organic acids and alcohols, and thus grows well under toxic concentrations of various compounds in the culture medium, potentially allowing for highly efficient compound production. (*R*)-citramalate is a raw material for methyl methacrylate and can be used as a metabolic intermediate in the biosynthesis of higher alcohols. (*R*)-citramalate is synthesized from pyruvate and acetyl-CoA. Unlike *Escherichia coli*, *S. cerevisiae* has organelles, and its intracellular metabolites are compartmentalized, preventing full use of intracellular acetyl-CoA. Therefore, in this study, to increase the amount of cytosolic acetyl-CoA for highly efficient production of (*R*)-citramalate, we inhibited the transport of cytosolic acetyl-CoA and pyruvate to the mitochondria. We also constructed a heterologous pathway to supply cytosolic acetyl-CoA. Additionally, we attempted to export (*R*)-citramalate from cells by expressing a heterologous dicarboxylate transporter gene. We evaluated the effects of these approaches on (*R*)-citramalate production and constructed a final strain by combining these positive approaches. The resulting strain produced 16.5 mM (*R*)-citramalate in batch culture flasks. This is the first report of (*R*)-citramalate production by recombinant *S. cerevisiae*, and the (*R*)-citramalate production by recombinant yeast achieved in this study was the highest reported to date.

## Highlights

• First report of (*R*)-citramalate production by engineered *Saccharomyces cerevisiae.*
• Deletion of either *POR1* or *YAT2* slightly improved (*R*)-citramalate production.
• PK and PTA improved the cytosolic acetyl-CoA supply pathway.
• SpMAE1 improved the secretion of (*R*)-citramalate in *S. cerevisiae.*
• Our approach increased the production of (*R*)-citramalate by more than four-fold.

## Abbreviations

6PGC: 6-phosphogluconate
6PGL: 6-phosphogluconolactone
AcAld: acetaldehyde
AcCoA: acetyl-CoA
Ace: acetate
ACH: acetone cyanohydrin
E4P: erythrose 4-phosphate
AcP: acetyl phosphate
F6P: fructose-6-phosphate
FBP: fructose-1,5-bisphosphate
G6P: glucose-6-phosphate
GAP: glyceraldehyde-3-phosphate
HPLC: high-performance liquid chromatography
MPC: mitochondrial pyruvate carrier
MMA: methyl methacrylate
OD600: optical density at 600 nm
ORF: open reading frame
PDH: pyruvate dehydrogenase
PK: phosphoketolase
PTA: phosphotransacetylase
R5P: ribose-5-phosphate
Ru5P: ribulose-5-phosphate
TAL: triacetic acid lactone
X5P: xylulose-5-phosphate

## 1 Introduction

(*R*)-citramalate is a dicarboxylic acid metabolite produced by microorganisms and plant cells. There is an industrial demand for (*R*)-citramalate as a precursor of methyl methacrylate (MMA). MMA is used as polymethyl methacrylate in various industrial products owing to its high clarity and impact resistance (Ali et al., 2015). The market economy of MMA grows every year and is expected to reach USD 8.16 billion by 2025 (Lebeau et al., 2020). The acetone cyanohydrin (ACH) route, which is the current method used for MMA synthesis, is an environmentally hazardous process that uses toxic reagents to produce many byproducts (Darabi Mahboub et al., 2018). However, the MMA synthesis method using (*R*)-citramalate as a precursor is attractive as an environmentally low-impact alternative to the ACH route because it uses a catalyst and water at high temperature and pressure, producing few byproducts and requiring no toxic reagents (Wu et al., 2021).

In a microbial metabolic reaction, 1 mol (*R*)-citramalate is synthesized from 1 mol pyruvate and 1 mol acetyl-CoA using citramalate synthase (EC 2.3.1.182). Several studies on the heterologous production of (*R*)-citramalate by microorganisms have been reported (Table 1), mostly using *Escherichia coli* (Wu and Eiteman, 2016; Parimi et al., 2017; Webb et al., 2018; Wu et al., 2020, 2023). However, the highly efficient production of (*R*)-citramalate by *E. coli* requires pH adjustment of the medium because of the low organic acid tolerance of *E. coli*. As the addition of neutralizers to the culture medium can increase the purification process after fermentation and product costs (Sauer et al., 2008; Nghiem et al., 2017; Bhagwat et al., 2021), it is desirable to realize an organic acid production process without the need for neutralization.

**Table 1:** Heterologous production of (*R*)-citramalate by engineered microorganisms.

Microorganism (Parent strain)	Genotype	Method	Duration [h]	Concentration [g/L]	Yield [g/g-glucose]	Reference
E. coli						
MEC499/pZE12-cimA (MG1655)	DgltA770:(FRT) DleuC778:(FRT) DackA778:Kan, pZE12-cimA	Fed-batch	132	46.5	0.63	Wu and Eiteman (2016)
MEC568/pZE12-cimA (MG1655)	ΔgltAΔleuCΔackA-ptaΔpoxB	Fed-batch	87	54.1	0.64	Parimi et al. (2017)
JW1 (BW25113)	ΔldhAΔpflB, pBAD24-mjcimA3.7	Fed-batch	65	82	0.48	Webb et al. (2018)
MEC626/pZE12-cimA (MG1655)	ΔleuCΔ(ackA-pta)ΔpoxBΔgltA:gltA(F383M)-Kan	Fed-batch	132	60	0.53	Wu et al. (2020)
I. orientalis						
SB814 and SB816 (SD108)	ΔURA3ΔLEU2, GFP-CimA-LEU (integrated by piggyBac transposase system)	Batch	48	2.0	0.06	Wu et al. (2023)
S. cerevisiae						
ICM-dYAT2-comp (YPH499)	ΔYAT2, X-4:cimA-SpMAE1-An_xfpk_Ck_pta-HIS3	Batch	96	2.5	0.03	This study

Yeast is a promising microorganism for (*R*)-citramalate production because they are more acid- and alcohol-tolerant than *E. coli* is and are also sufficiently robust to withstand repeated fermentation and industrial use. Wu et al. (2023) randomly integrated a citramalate synthase gene (*cimA*) from *Methanocaldococcus jannaschii* into the chromosome of *Issatchenkia orientalis*, a low-pH-tolerant non-model yeast, using a piggyBac transposon system and successfully produced 2.0 g/L (*R*)-citramalate by batch fermentation.

Although the budding yeast, *Saccharomyces cerevisiae*, has been well studied as a host for the heterologous production of useful compounds, to the best of our knowledge, there are no reports on its use in the production of (*R*)-citramalate as a target compound, and there are only a few reports on alcohol production using (*R*)-citramalate as an intermediate (Shi et al., 2016; Nishimura et al., 2018).

As *S. cerevisiae* is not a natural producer of (*R*)-citramalate, it must heterologously express *cimA*. If *cimA* is expressed in the cytosol, the supply of both cytosolic pyruvate and cytosolic acetyl-CoA, which are substrates for (*R*)-citramalate synthesis, is important. However, the amount of acetyl-CoA in the yeast cytosol is thought to be less than that in *E. coli* because of the compartmentalization of intracellular acetyl-CoA by organelles. In previous research, the cytosolic acetyl CoA concentration in *E. coli* was estimated to range from 20 to 600 μM (Takamura and Nomura, 1988). In contrast, intracellular acetyl-CoA concentration in *S. cerevisiae* was estimated to be ∼30 μM, and one-third of the total acetyl-CoA pool was presumed to accumulate in the mitochondria. This suggests that the cytosolic acetyl-CoA concentration is lower than that in *E. coli* (Weinert et al., 2014). Organelles complicate metabolic engineering for high production from acetyl-CoA in the yeast cytosol, which is one of the fundamental challenges in the metabolic engineering of yeast (Krivoruchko et al., 2015). Cytosolic pyruvate, which is synthesized via glycolysis, can be converted by *S. cerevisiae* into acetyl-CoA using endogenous pyruvate decarboxylase, acetaldehyde dehydrogenase (ALD), and acetyl-CoA synthetase. To increase the amount of cytosolic acetyl-CoA in *S. cerevisiae*, in addition to enhancing the endogenous cytosolic acetyl-CoA synthesis pathway, a common strategy is to construct heterologous pathways for acetyl-CoA supply (van Rossum et al., 2016). For example, there are pathways that can convert acetaldehyde to acetyl-CoA via the pyruvate dehydrogenase (PDH) complex from *E. coli* or *Enterococcus faecalis* (van Rossum et al., 2016). As the endogenous and heterologous pathways listed above require NAD(P) or ATP, an appropriate pathway must be selected that does not compete with the cofactor demand associated with target compound synthesis.

In addition, inhibition of cytosolic acetyl-CoA and pyruvate transport to organelles may be effective in increasing the production of cytosolic acetyl-CoA. Cardenas and Da Silva (2016) individually disrupted seven genes involved in the transport of acetyl-CoA and pyruvate to the mitochondria and evaluated their effects on triacetic acid lactone (TAL) production in *S. cerevisiae*. They found that disruption of the mitochondrial pyruvate carrier (MPC) subunit (MPC2), the E1 alpha subunit of the PDH complex (PDA), mitochondrial porin (POR2), and carnitine acetyltransferase (YAT2) increased TAL production. However, reports on the inhibition of cytosolic acetyl-CoA and pyruvate transport to organelles are lacking, and further investigation is needed to determine whether this approach universally contributes to efficient bioproduction using cytosolic acetyl-CoA as a substrate.

**Fig. 1:**
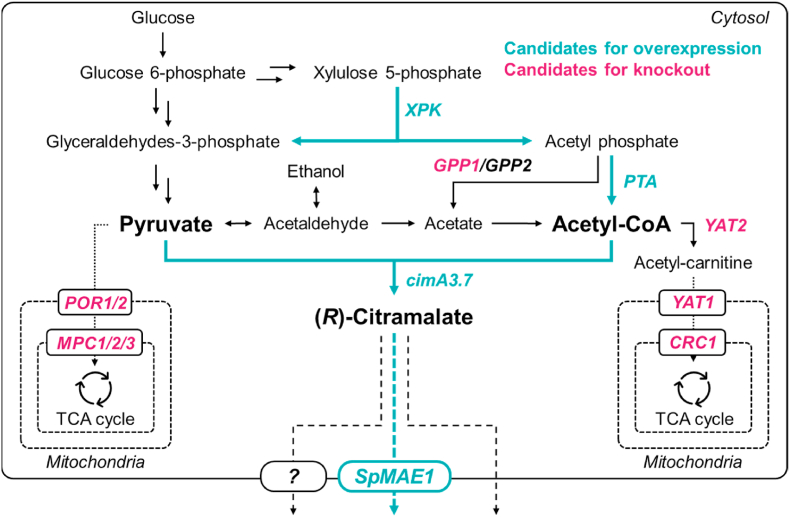
(*R*)-citramalate production pathway in *S*. *cerevisiae*.

In this study, four independent experiments were performed to heterologously produce (*R*)-citramalate by *S. cerevisiae* (Fig. 1), which is synthesized from cytosolic pyruvate and acetyl-CoA. First, the transport of pyruvate and acetyl-CoA from the cytosol to the mitochondria was inhibited by gene deletion. Second, a heterologous pathway was introduced to enhance the cytosolic acetyl-CoA supply. Third, a heterologous malate transporter gene was evaluated for improved (*R*)-citramalate secretion. Fourth, the effects of different copy numbers of *cimA* on (*R*)-citramalate production were evaluated. Finally, we aimed to develop a yeast strain with high (*R*)-citramalate production by combining the results obtained from these four independent experiments. This is the first report of (*R*)-citramalate production by *S. cerevisiae*, and our results could contribute to the development of metabolic engineering not only for (*R*)-citramalate production, but also for heterologous production from cytosolic pyruvate and acetyl-CoA in *S. cerevisiae*.

Overview of the yeast metabolic pathway for (*R*)-citramalate production. Black arrows represent native pathways, blue arrows the heterologous pathways, and red genes the candidates for gene deletion targeted in this study.

## 2 Materials and methods

### 2.1 Strains and media

The yeast strains used in this study are listed in Table 2. The *E. coli* NovaBlue competent cells (Novagen, Cambridge, MA, USA) were used for gene cloning. LB medium (BD Difco, Franklin Lakes, NJ, USA) supplemented with 100 μg/mL ampicillin sodium salt was used to culture *E. coli*. YPD (10 g/L yeast extract, 20 g/L peptone, and 20 g/L glucose), YPG (10 g/L yeast extract, 20 g/L peptone, and 20 g/L glycerol), SD (6.7 g/L yeast nitrogen base without amino acids [BD Difco] and 20 g/L glucose), or SG (6.7 g/L yeast nitrogen base without amino acids, 20 g/L galactose, and 10 g/L raffinose) media were used to culture yeast in test tubes. SD and SG media were supplemented with the appropriate amino acids. In flask cultures, the glucose concentration in SD or YPD medium was changed to 50 or 100 g/L, respectively.

**Table 2:** Yeast strains used in this study.

Strain	Genotype	Reference
*S. cerevisiae* YPH499	*MATa*, *ura3-52*, *leu2-*Δ*1*, *lys2-801*, *his-*Δ*200*, *trp1-*Δ*63*, *ade2-101*	ATCC
YPH499dMPC1	YPH499, Δ*MPC1*	This study
YPH499dMPC2	YPH499, Δ*MPC2*	This study
YPH499dMPC3	YPH499, Δ*MPC3*	This study
YPH499dPOR1	YPH499, Δ*POR1*	This study
YPH499dPOR2	YPH499, Δ*POR2*	This study
YPH499dYAT1	YPH499, Δ*YAT1*	This study
YPH499dYAT2	YPH499, Δ*YAT2*	This study
YPH499dCRC1	YPH499, Δ*CRC1*	This study
ECM	YPH499, pEUsp-cimA3.7	This study
CCM	YPH499, pCUsp-cimA3.7	This study
ICM	YPH499, X-4:*pPGK1-cimA3.7-tPRM9*	This study
ECM-dMPC1	ECM, Δ*MPC1*	This study
ECM-dMPC2	ECM, Δ*MPC2*	This study
ECM-dMPC3	ECM, Δ*MPC3*	This study
ECM-dPOR1	ECM, Δ*POR1*	This study
ECM-dPOR2	ECM, Δ*POR2*	This study
ECM-dYAT1	ECM, Δ*YAT1*	This study
ECM-dYAT2	ECM, Δ*YAT2*	This study
ECM-dCRC1	ECM, Δ*CRC1*	This study
ECM-dGPP1	ECM, Δ*GPP1*	This study
ECM-dPOR1YAT2	ECM, Δ*POR1*, Δ*YAT2*	This study
ECM-pGK423	ECM, pGK423,	This study
ECM-pGK423-pGK424	ECM, pGK423, pGK424	This study
ECM-BsAn	ECM, pGK423-Bs_pta, pGK424-An_xfpk	This study
ECM-CkAn	ECM, pGK423-Ck_pta, pGK424-An_xfpk	This study
ECM-dGPP1-BsAn	ECM-dGPP1, pGK423-Bs_pta, pGK424-An_xfpk	This study
ECM-dGPP1-BsBb	ECM-dGPP1, pGK423-Bs_pta, pGK424-Bb_xfpk	This study
ECM-dGPP1-BsBl	ECM-dGPP1, pGK423-Bs_pta, pGK424-Bl_xfspk	This study
ECM-dGPP1-BsLm	ECM-dGPP1, pGK423-Bs_pta, pGK424-Lm_xfpk	This study
ECM-dGPP1-CkAn	ECM-dGPP1, pGK423-Ck_pta, pGK424-An_xfpk	This study
ECM-dGPP1-CkBb	ECM-dGPP1, pGK423-Ck_pta, pGK424-Bb_xfpk	This study
ECM-dGPP1-CkBl	ECM-dGPP1, pGK423-Ck_pta, pGK424-Bl_xfspk	This study
ECM-dGPP1-CkLm	ECM-dGPP1, pGK423-Ck_pta, pGK424-Lm_xfpk	This study
ECM-SpMAE1	ECM, pGK423-SpMAE1	This study
ICM-comp	YPH499, X-4:*pPGK1-cimA3.7-tPRM9-pTEF1-SpMAE1-tDIT1-pTPI1-An_xfpk-tHXT7-pTDH3-Ck_pta-tADH1-pHIS3-HIS3-tHIS3*	This study
ICM-dPOR1-comp	ICM-comp, Δ*POR1*	This study
ICM-dYAT2-comp	ICM-comp, Δ*YAT2*	This study
ICM-dPOR1dYAT2-comp	ICM-comp, Δ*POR1*Δ*YAT2*	This study

### 2.2 Plasmid construction

The plasmids used in this study are listed in Table 3. The pRS316 and pGAL1-Cas9-tADH1-pGAL1-2BsaI-sgRNAFE (empty)-HDV-tCYC1-CU (Okada et al., 2021) were purchased from the National BioResource Project (NBRP; Japan). The primer pairs used for PCR are listed in Supplementary Table 1. DNA fragments were assembled using NEBuilder (New England Biolabs, Ipswich, MA, USA). Detailed methods for plasmid construction are described in the Supplementary Data.

**Table 3:** Plasmids used in this study.

Plasmid	Description	Reference
pRS316	Yeast centromeric vector containing *CEN/ARS*, *URA3* marker	NBRP
pGK423	Yeast episomal vector containing p*PGK1*, t*PGK1*, *2 μ* origin, *HIS3* marker	Ishii et al. (2009)
pGK424	Yeast episomal vector containing p*PGK1*, t*PGK1*, *2 μ* origin, *TRP1* marker	Ishii et al. (2009)
pGK426	Yeast episomal vector containing p*PGK1*, t*PGK1*, *2 μ* origin, *URA3* marker	Ishii et al. (2009)
pGAL1-Cas9-tADH1-pGAL1-2BsaI-sgRNAFE (empty)-HDV-tCYC1-CU	p*GAL1-Cas9-*t*ADH1*-p*GAL1*-2BsaI-*sgRNAFE* (empty)-*HDV*-t*CYC1*-*CU*, *ARS/CEN*, *URA3* marker, backbone vector for gene deletion	Okada et al. (2021)
pUCas9	pGAL1-Cas9-tADH1-pGAL1, ARS/CEN, URA3 marker	This study
pgRNA	pGAL1-2BsaI-sgRNAFE (20 bp upstream of PAM in chromosome X-4 site)-HDV-tCYC1-CU, no replicant origin and selection marker genes for yeast	This study
pgRNA_ChX-4	pGAL1-2BsaI-sgRNAFE (empty)-HDV-tCYC1-CU, no replicant origin and selection marker genes for yeast	This study
pCUsp	Yeast centromeric vector containing p*PGK1*, t*PRM9*, *CEN/ARS*, *URA3* marker	This study
pEUsp	Yeast episomal vector containing p*PGK1*, t*PRM9*, *2 μ* origin, *URA3* marker	This study
pIntChX-4	Yeast integrative vector containing homologous region to chromosome X-4 site, *URA3* marker	This study
pCRISPR-MPC1del	p*GAL1-Cas9-*t*ADH1*-p*GAL1*-2BsaI-*sgRNAFE* (20 bp upstream of PAM in MPC1 ORF)-*HDV*-t*CYC1*-*CU*, *ARS/CEN*, *URA3* marker	This study
pCRISPR-MPC2del	p*GAL1-Cas9-*t*ADH1*-p*GAL1*-2BsaI-*sgRNAFE* (20 bp upstream of PAM in MPC2 ORF)-*HDV*-t*CYC1*-*CU*, *ARS/CEN*, *URA3* marker	This study
pCRISPR-MPC3del	p*GAL1-Cas9-*t*ADH1*-p*GAL1*-2BsaI-*sgRNAFE* (20 bp upstream of PAM in MPC3 ORF)-*HDV*-t*CYC1*-*CU*, *ARS/CEN*, *URA3* marker	This study
pCRISPR-POR1del	p*GAL1-Cas9-*t*ADH1*-p*GAL1*-2BsaI-*sgRNAFE* (20 bp upstream of PAM in POR1 ORF)-*HDV*-t*CYC1*-*CU*, *ARS/CEN*, *URA3* marker	This study
pCRISPR-POR2del	p*GAL1-Cas9-*t*ADH1*-p*GAL1*-2BsaI-*sgRNAFE* (20 bp upstream of PAM in POR2 ORF)-*HDV*-t*CYC1*-*CU*, *ARS/CEN*, *URA3* marker	This study
pCRISPR-YAT1del	p*GAL1-Cas9-*t*ADH1*-p*GAL1*-2BsaI-*sgRNAFE* (20 bp upstream of PAM in YAT1 ORF)-*HDV*-t*CYC1*-*CU*, *ARS/CEN*, *URA3* marker	This study
pCRISPR-YAT2del	p*GAL1-Cas9-*t*ADH1*-p*GAL1*-2BsaI-*sgRNAFE* (20 bp upstream of PAM in YAT2 ORF)-*HDV*-t*CYC1*-*CU*, *ARS/CEN*, *URA3* marker	This study
pCRISPR-CRC1del	p*GAL1-Cas9-*t*ADH1*-p*GAL1*-2BsaI-*sgRNAFE* (20 bp upstream of PAM in CRC1 ORF)-*HDV*-t*CYC1*-*CU*, *ARS/CEN*, *URA3* marker	This study
pCRISPR-GPP1del	p*GAL1-Cas9-*t*ADH1*-p*GAL1*-2BsaI-*sgRNAFE* (20 bp upstream of PAM in GPP1 ORF)-*HDV*-t*CYC1*-*CU*, *ARS/CEN*, *URA3* marker	This study
pGK423-Bs_pta	pGK423 expressing *Bs_pta*	This study
pGK423-Ck_pta	pGK423 expressing *Ck_pta*	This study
pGK423-SpMAE1	pGK423 expressing *SpMAE1*	This study
pGK424-An_xfpk	pGK424 expressing *An_xfpk*	This study
pGK424-Bb_xfpk	pGK424 expressing *Bb_xfpk*	This study
pGK424-Bl_xfspk	pGK424 expressing *Bl_xfspk*	This study
pGK424-Lm_xfpk	pGK424 expressing *Lm_xfpk*	This study
pEUsp-cimA3.7	pEUsp expressing *cimA3.7*	This study
pCUsp-cimA3.7	pCUsp expressing *cimA3.7*	This study
pIntChX-4_cimA3.7	pIntChX-4, p*PGK1*-*cimA3.7*-t*PGK1*	This study
pChX-4_up_cimA3.7	500 bp homologous to X-4 site, pPGK1-cimA3.7-tPRM9, used for gene insertion into X-4 site	This study
pSpMAE1	pTEF1-SpMAE1-tDIT1, used for gene insertion into X-4 site	This study
pAn_xfpk	pTPI1-An_xfpk-tHXT7, used for gene insertion into X-4 site	This study
pCk_pta	pTDH3-Ck_pta-tADH1, used for gene insertion into X-4 site	This study
pHIS3_ChX-4_down	*HIS3* marker, 500 bp homologous to X-4 site, used for gene insertion into X-4 site	This study

### 2.3 Yeast transformation

Yeast transformation was performed as previously described (Chen et al., 1992). A yeast strain was grown for 1–3 days to steady state and centrifuged (5000×*g*, 20 °C) for 1 min to remove the supernatant. For gene insertion only, a yeast strain was incubated in YPG medium for 2 h before transformation. The cell pellet was then washed once with 100 μL one-step buffer (40% [w/v] PEG4000, 0.1 M DTT, and 0.2 M lithium acetate). The cell pellet was then resuspended in 100 μL one-step buffer, and 10 μL of 10 mg/mL carrier DNA (ice-cooled after incubation at 95 °C for 5 min) and DNA solution added thereafter. The cell suspension was incubated at 42 °C for 45 min and maintained on ice for 5 min. Afterwards, it was appropriately diluted with sterile water, and 100 μL cell suspension spread onto appropriate selection medium and kept at 30 °C for 2–7 days until colony formation.

### 2.4 Gene deletion and insertion

Gene deletion was performed as previously described (Okada et al., 2021). Detailed methods for gene deletion and insertion are described in the Supplementary Data. Gene deletion was achieved by completely removing the open reading frame (ORF) from the gene of interest. For insertion of only the *cimA3.7* expression cassette into the chromosome X-4 site (Mikkelsen et al., 2012), 1.5 μg pIntChX-4_cimA3.7 was digested with *Asc*I (37 °C for 2 h) and used for transformation. To insert multiple gene expression cassettes, five DNA fragments were prepared via PCR using the five plasmids, pChX-4_up_cimA3.7, pSpMAE1, pAn_xfpk, pCk_pta, and pHIS3_ChX-4_down, as templates, with the corresponding primers (Supplementary Table 1) for yeast transformation. Recombinants were confirmed through colony direct PCR.

### 2.5 Yeast culture

In test tube cultures, yeast cells were cultured for 1–3 days at 30 °C in SD medium. Thereafter, the amount of cells required for inoculation with an optical density at 600 nm (OD600) of 0.05 or 0.3 was calculated, the broth centrifuged (20 °C, 5000×*g*) for 1 min, and the supernatant removed to collect the cells. Then, the cells were inoculated in 5 mL SD medium with an OD600 of 0.05 or 0.3 and cultured at 30 °C with agitation (180 rpm).

Flask cultures were performed in 40 mL YPD or SD medium containing 50 g/L or 100 g/L glucose at 30 °C with agitation (150 rpm). Fermentation was initiated by inoculating cells that were pre-cultured and collected, as described for the test tube cultures, with an initial OD600 of 0.2 or 0.5.

### 2.6 Analytical methods

Cell growth was monitored by measuring the OD600 of the culture diluted in 10 mM EDTA with a UV-1280 spectrophotometer (Shimadzu, Kyoto, Japan). Moreover, the culture was centrifuged (5000×*g*, 20 °C) for 1 min, and the supernatant kept at −30 °C and thawed for subsequent pH measurements and high-performance liquid chromatography (HPLC) analysis.

The pH of the culture supernatant was measured using LAQUAtwin (HORIBA, Kyoto, Japan). The concentrations of (*R*)-citramalate and pyruvate in the culture supernatant were determined according to a previously described HPLC method (Noda et al., 2017) using an organic acid analysis system (Shimadzu) consisting of an HPLC instrument equipped with a Shim-pack SCR-102H column. Besides, concentrations of the other compounds present in the culture supernatant were determined using an HPLC system equipped with an Aminex HPX-87H column (Bio-Rad, Hercules, CA, USA). The column was operated at 55 °C with a flow rate of 0.5 mL/min, and RID-20A (Shimadzu) used as a detector. The mobile phase used consisted of 5 mM H_2_SO_4_.

## 3 Results

### 3.1 Expression of citramalate synthase in yeast

**Fig. 2:**
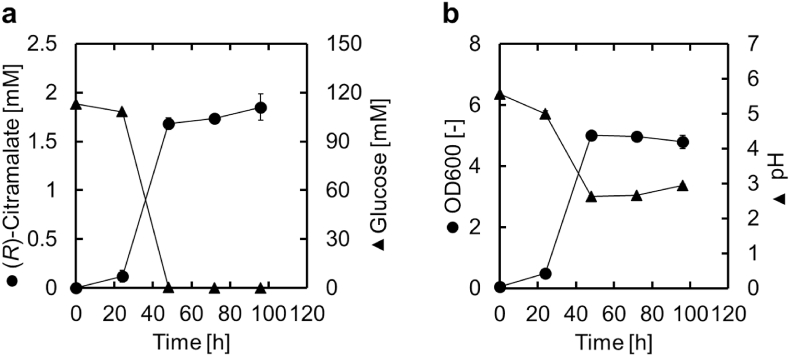
Heterologous production of (*R*)-citramalate by a *S*. *cerevisiae* strain expressing *cimA3.7*.

First, the citramalate synthase gene (*cimA3.7*), a mutant enzyme from *M. jannaschii* with greatly enhanced activity at 30 °C (Atsumi and Liao, 2008), was expressed in *S. cerevisiae* to evaluate its ability to facilitate (*R*)-citramalate production. The recombinant *S. cerevisiae* ECM strain, obtained by transforming *S. cerevisiae* YPH499 with the 2*μ* plasmid expressing *cimA3.7* (pEUsp-cimA3.7) was cultured in test tubes, and the concentration of (*R*)-citramalate in the supernatants measured via HPLC. ECM produced up to 1.85 mM (274.0 mg/L) (*R*)-citramalate during culture (Fig. 2a). This indicated that the *S. cerevisiae* strain expressing *cimA3.7* was able to produce and excrete (*R*)-citramalate without the need to engineer transporters. The concentration of (*R*)-citramalate continued to increase slightly after glucose depletion, and the pH of the culture remained below 3. The high acid tolerance of yeast capable of producing (*R*)-citramalate under acidic conditions provides an advantage in the non-neutralization process. In the following experiments, ECM was used as the base strain.

Production of (*R*)-citramalate by *S*. *cerevisiae* ECM, a yeast strain expressing *cimA3.7* on a *2μ* plasmid, and its growth. (a-b) Time courses of the (a) (*R*)-citramalate and glucose concentrations and (b) the OD600 and pH in culture. The initial OD600 value of culture was 0.05. All data are presented as averages of three independent experiments. Error bars represent standard deviations.

### 3.2 Gene knockout for the improvement of citramalate production

**Fig. 3:**
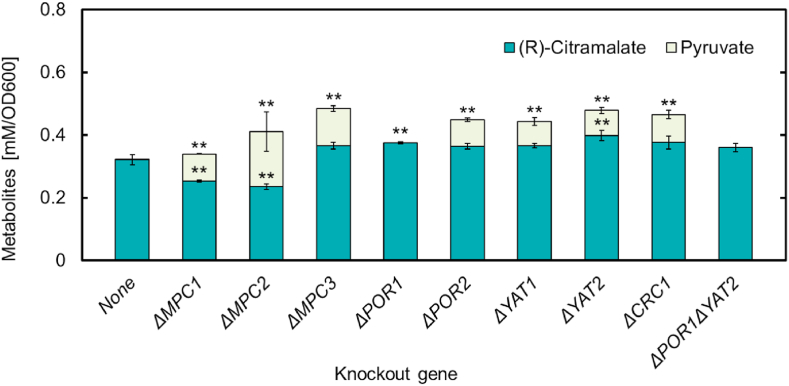
Production of (*R*)-citramalate and pyruvate by gene-knockout strains (*R*)-Citramalate and pyruvate production per OD600 value by eight gene-knockout strains at 96 h. The initial OD600 values of culture were 0.3. All data are presented as averages of three independent experiments. Error bars represent standard deviations. Asterisks indicate significant differences when compared to the value of “None” at the same time point (Student's t-test; **P < 0.01).

Cytosolic pyruvate and acetyl-CoA, which are substrates for (*R*)-citramalate synthesis, are both partially transported into the mitochondria to meet the demand for acetyl-CoA. As this transport could not be completely prevented, eight genes involved in the transport of pyruvate and acetyl-CoA to the mitochondria were individually deleted to investigate the possibility of increasing (*R*)-citramalate production. Eight knockout strains were transformed with pEUsp-cimA3.7 to construct eight recombinant strains named ECM-dX (where X corresponds to the knockout gene). As shown in Fig. 3, two of the eight strains, ECM-dPOR1 and ECM-dYAT2, showed a slight increase in (*R*)-citramalate production per OD600 value compared to that of ECM. We confirmed that these two strains were significantly different compared to ECM production by student's t-test (P < 0.01) (Fig. 3). Unlike seven other knockout strains, ECM-dPOR1 showed no extracellular pyruvate at 96 h Δ*POR1* and Δ*YAT2* were targeted to suppress pyruvate and acetyl-CoA transport, respectively. However, the pyruvate accumulation of ECM-dPOR1 did not improve compared to that of ECM. The other strains showed no improvement in (*R*)-citramalate production.

YAT2 is a cytosolic carnitine acetyltransferase responsible for the first step of the carnitine shuttle, which converts acetyl-CoA to acetyl-carnitine (Swiegers et al., 2001; Franken et al., 2008). POR1 is a mitochondrial porin, which is the protein of the mitochondrial outer membrane required for maintaining mitochondrial osmotic stability and membrane permeability (Lee et al., 1998; Sánchez et al., 2001). We constructed the double gene-knockout strain, ECM-dPOR1dYAT2, to examine the synergistic effects of these two gene deletions on (*R*)-citramalate production. However, this strain produced the same amount of (*R*)-citramalate as that of ECM and showed no significant increase in production (Fig. 3). This suggests that transport inhibition alone is not sufficient to increase cytosolic acetyl-CoA levels, and that it is important to either enhance the endogenous pathway or construct a heterologous pathway.

### 3.3 Phosphoketolase/phosphotransacetylase pathway evaluation

**Fig. 4:**
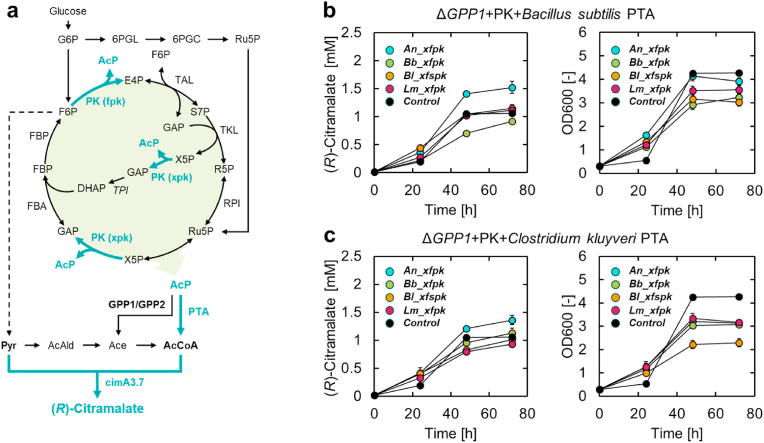
Construction of a phosphoketolase/phosphotransacetylase pathway in yeast (a) Yeast metabolic pathway with cimA3.7, phosphoketolase (PK), and phosphotransacetylase (PTA). Black colors represent native pathways, metabolites, and enzymes, whereas blue colors represent heterologous pathways, metabolites, and enzymes. PK has two enzymatic activities (xpk and fpk) and synthesizes 3 mol acetyl phosphate (AcP) from 1 mol glucose. (b–c) Time course of (*R*)-citramalate production and growth of strains expressing PK and PTA genes. All strains in (b) expressed the *B*. *subtilis* PTA gene (*Bs_pta*), and those in (c) expressed the *C*. *kluyveri* PTA gene (*Ck_pta*). The initial OD600 values of culture were 0.3. All data are presented as averages of three independent experiments. Error bars represent standard deviations. Abbreviations indicate the following metabolites: 6PGC, 6-phosphogluconate; 6PGL, 6-phosphogluconolactone; AcAld, acetaldehyde; AcCoA, acetyl-CoA; Ace, acetate; AcP, acetyl phosphate; DHAP, dihydroxyacetone phosphate; E4P, erythrose 4-phosphate; F6P, fructose-6-phosphate; FBP, fructose-1,5-bisphosphate; G6P, glucose-6-phosphate; GAP, glyceraldehyde-3-phosphate; R5P, ribose-5-phosphate; Ru5P, ribulose-5-phosphate; S7P, sedoheptulose-7-phosphate; X5P, xylulose-5-phosphate; Pyr, pyruvate. (For interpretation of the references to color in this figure legend, the reader is referred to the Web version of this article.)

The heterologous expression of phosphoketolase (PK) and phosphotransacetylase (PTA) in *S. cerevisiae* can be used to construct a heterologous pathway (PK/PTA pathway) to synthesize acetyl-CoA from fructose-6-phosphate (F6P), xylulose-5-phosphate (X5P), or ribulose-5-phosphate via acetyl-phosphate (Fig. 4a). The advantage of this pathway is that it can synthesize 3 mol acetyl-CoA from 1 mol glucose without carbon loss due to CO_2_ (van Rossum et al., 2016). To construct an appropriate PK/PTA pathway, we evaluated four different PKs from different organisms, namely *Aspergillus nidulans*, *Bifidobacterium breve*, *Bifidobacterium longum*, and *Leuconostoc mesenteroides* (*An_xfpk*, *Bb_xfpk*, *Bl_xfpk*, and *Lm_xfspk*, respectively), and two PTAs from *Bacillus subtilis* and *Clostridium kluyveri* (*Bs_pta* and *Ck_pta*, respectively) (Sonderegger et al., 2004; Bergman et al., 2016; Hellgren et al., 2020). As acetyl-phosphate can be converted to acetate by endogenous GPP1 and GPP2 in *S. cerevisiae*, and GPP1 is thought to be the main intracellular enzyme involved in this reaction (Bergman et al., 2016), we used the *GPP1* knockout strain, ECM-dGPP1, as the host strain. Eight recombinant strains expressing different PKs and PTAs were constructed and compared for the production of (*R*)-citramalate. When comparing the production at 72 h (Fig. 4b and c), An_xfpk was the most suitable for (*R*)-citramalate production, regardless of the PTA gene used. The OD600 value in the stationary phase of all recombinant strains expressing the PK/PTA genes was lower than that of the control strain with the empty vectors. For the two strains ECM-BlBs and ECM-BlCk expressing *Bl_xfspk*, a small amount of glucose remained in the medium at 72 h (data not shown).

**Fig. 5:**
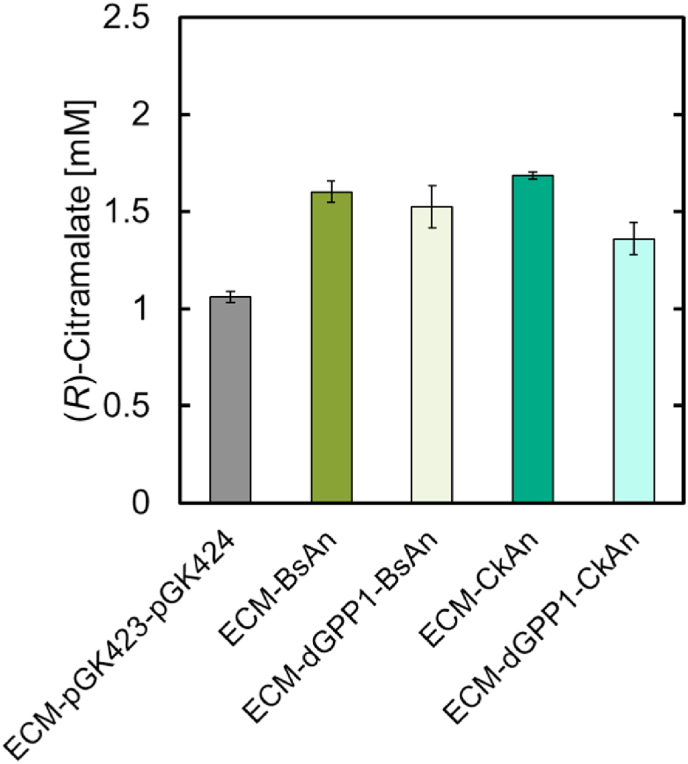
Effect of *GPP1* expression on (*R*)-citramalate production in strains with a phosphoketolase/phosphotransacetylase pathway (*R*)-citramalate production in strains expressing PK and PTA genes at 72 h. ECM-BsAn and ECM-CkAn were strains that expressed endogenous *GPP1*, whereas ECM-dGPP1-BsAn and ECM-dGPP1-CkAn were strains whose *GPP1* was deleted. ECM-pGK423-pGK424 was the control strain that did not express PK and PTA genes. All data are presented as averages of three independent experiments. Error bars represent standard deviations.

Next, we evaluated the effect of *GPP1* on (*R*)-citramalate production by culturing ECM-dGPP1-AnBs, ECM-dGPP1-AnCk, ECM-AnBs, and ECM-AnCk cells. As shown in Fig. 5, the deletion of *GPP1* had no positive effect on (*R*)-citramalate production. ECM-AnCk showed a 1.6-fold increase in (*R*)-citramalate production at 72 h compared to that of strains without the PK/PTA pathway. This suggests that the PK/PTA pathway functions in yeast cells and improves the supply of cytosolic acetyl-CoA. Moreover, the deletion of *GPP1* did not improve (*R*)-citramalate production, suggesting that the reaction from acetyl-phosphate to acetyl-CoA may be rate-limiting, and that by increasing flux to the endogenous acetyl-CoA pathway via acetate or expressing a more highly active PTA could provide higher acetyl-CoA levels.

### 3.4 Expression of a heterologous malate transporter

**Fig. 6:**
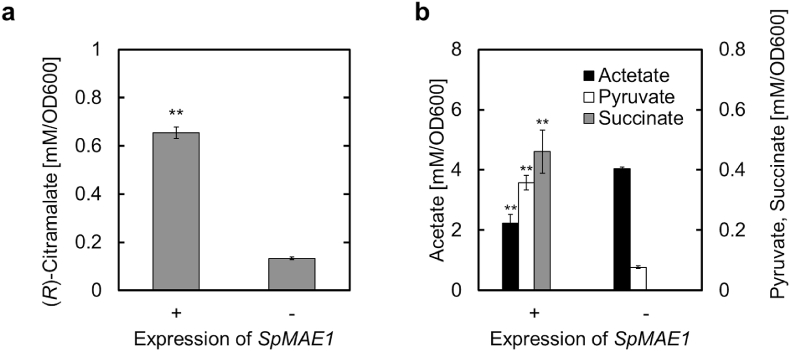
Expression of a heterologous malate transporter.

In the ECM culture, we confirmed that the (*R*)-citramalate synthesized in *S. cerevisiae* cells was secreted extracellularly. However, if this extracellular transport is insufficient and some (*R*)-citramalate accumulates intracellularly, it would be necessary to improve its extracellular transport. In *S. cerevisiae*, the endogenous transporter that greatly improves the secretion of dicarboxylic acids, such as (*R*)-citramalate, is unknown. Therefore, we attempted to improve the secretion of (*R*)-citramalate, a 5-carbon dicarboxylic acid, using a malate transporter from *Schizosaccharomyces pombe* (SpMAE1), which has been reported to greatly improve 4-carbon dicarboxylic acid secretion in *S. cerevisiae* (Darbani et al., 2019). As shown in Fig. 6a, *SpMAE1* expression increased (*R*)-citramalate production per OD600 by 4.9-fold compared to that of the *SpMAE1* non-expressing strain, ECM-pGK423, indicating that *SpMAE1* expression is effective for the secretion of (*R*)-citramalate. However, because the extracellular concentrations of pyruvate and succinate were also increased by *SpMAE1* expression (Fig. 6b), SpMAE1 may be responsible for broad acid transport and could promote the secretion of organic acids necessary for cell activity. ECM-SpMAE1 cells showed poorer growth than ECM-pGK423 cells did (Supplementary Fig. 1a). However, this growth inhibition was not fatal and effectively promoted (*R*)-citramalate secretion. Incidentally, ECM-pGK423 showed a significant decrease in (*R*)-citramalate concentration compared to the parental ECM strain (Supplementary Fig. 1b). Although the reason for this result is currently unknown, ECM-SpMAE1 still showed a 1.4-fold higher concentration compared to ECM, the parent strain of ECM-pGK423 (Supplementary Fig. 1b). From above results, we concluded that the expression of *SpMAE1* provides a benefit in (*R*)-citramalate production.

Expression of the malate transporter gene, *MAE1*, from *S*. *pombe* (*SpMAE1*) in *S*. *cerevisiae* ECM. (a) Comparison of (*R*)-citramalate production per OD600 value between strains expressing *SpMAE1* and those that did not at 96 h. (b) Production of three acid metabolites per OD600 value between the two strains. All data are presented as averages of three independent experiments. Error bars represent standard deviations. Asterisks indicate significant differences as determined by Student's t-test (**P < 0.01).

### *3.5* *Effect of* cimA3.7 *copy number on (*R*)-citramalate production*

Appropriate gene expression is important for establishing efficient metabolic pathways for bioproduction (Yamada et al., 2017). Here, we investigated the effect of *cimA3.7* copy number on (*R*)-citramalate production. The two recombinant strains expressing *cimA3.7* on a centromeric plasmid or chromosome X-4 site were named CCM and ICM, respectively. The X-4 site (Supplementary Table 5) was decided by referencing a chromosomal locus for gene integration and high gene expression in *S. cerevisiae* EN.PK113-7D (Mikkelsen et al., 2012). ECM, CCM, and ICM were cultured in test tubes, and their culture profiles obtained. Comparing the (*R*)-citramalate concentration [mM] at 96 h (Table 4), that of ECM, CCM, and ICM were 1.85 mM, 1.78 mM, and 2.22 mM, respectively, whereas the production [mM/OD600] was not different among the strains. These results indicate that the production capacity per cell remained unchanged, and higher cell concentrations lead to higher (*R*)-citramalate production in ICM compared to that in ECM and CCM. We speculate that this was likely due to the burden on the cells associated with plasmid retention and high gene expression, which resulted in reduced growth.

**Table 4:** Effect of *cimA3.7* copy number on (*R*)-citramalate production.

Strain	Vector type	Copy number	(*R*)-Citramalate production at 96 h	
			[mM]	[mM/OD600]
ECM	Episomal	High	1.85 ± 0.14	0.39 ± 0.01
CCM	Centromeric	Low	1.78 ± 0.11	0.34 ± 0.02
ICM	Integrative	Low (one copy)	2.22 ± 0.10	0.38 ± 0.02

All data represent the means ± SD of biological triplicates.

### *3.6* *Overproduction of (*R*)-citramalate by engineered yeast*

**Fig. 7:**
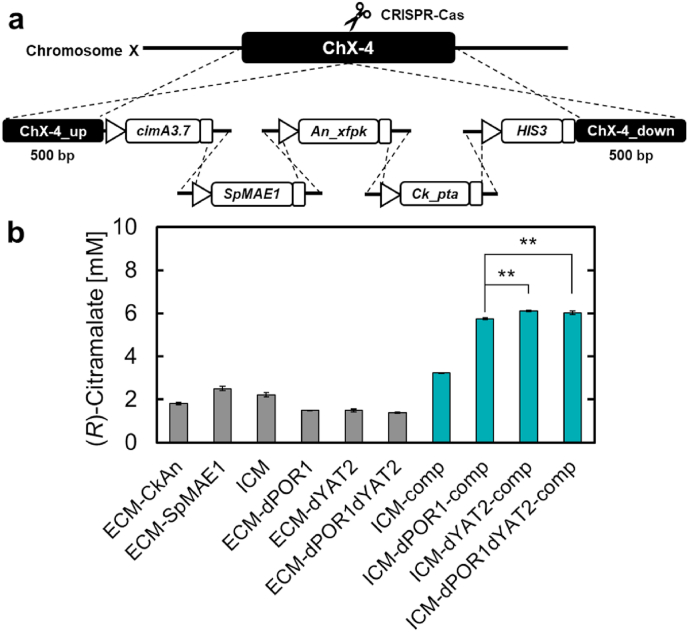
Overproduction of (*R*)-citramalate by engineered yeast in test tubes.

Finally, we combined our findings to construct recombinant yeasts with high (*R*)-citramalate production by integrating *cimA3.7*, *SpMAE1*, *An_xfpk*, and *Ck_pta* into the X-4 site using the CRISPR-Cas system (Fig. 7a). The resulting strains were cultured in test tubes. Fig. 7b shows the data on (*R*)-citramalate production at 96 h for the recombinant strains constructed thus far. ICM-comp, a transformant of YPH499, produced 3.22 mM (476.9 mg/L) (*R*)-citramalate at 96 h, which was 1.78-, 1.29-, and 1.45-fold higher than that of the PK/PTA pathway (ECM-AnCk), *SpMAE1*-expressing (ECM-SpMAE1), and chromosomal *cimA3.7*-expressing (ICM) strains, respectively. However, the transformants, ICM-dPOR1-comp, ICM-dYAT2-comp, and ICM-dPOR1dYAT2-comp, all produced approximately 6 mM (888.7 mg/L) (*R*)-citramalate, suggesting a synergistic effect of the four approaches. This synergistic effect resulted in a 4.39-fold increase in the Δ*POR1*Δ*YAT2* strain (ECM-dPOR1dYAT2 vs. ICM-dPOR1dYAT2-comp), a 4.11-fold increase in the Δ*YAT2* strain (ECM-dYAT2 vs. ICM-dYAT2-comp), and a 3.82-fold increase in (*R*)-citramalate production in the Δ*POR1* strain (ECM-dPOR1 vs. ICM-dPOR1-comp), respectively. The (*R*)-citramalate production of ICM-dPOR1dYAT2-comp was increased compared to that of ICM-dPOR1-comp, whereas there was no significant difference observed between ICM-dPOR1dYAT2-comp and ICM-dYAT2-comp; thus, the synergistic effect of Δ*POR1*Δ*YAT2* was not observed.

**Fig. 8:**
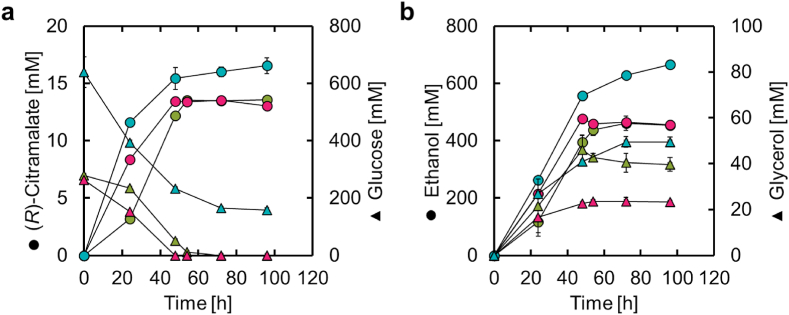
Batch fermentation profiles of ICM-dYAT2-comp Fermentation profiles of ICM-dYAT2-comp in batch fermentations using flasks. Green, red, and blue indicate SD50 (50 g/L glucose), YPD50 (50 g/L glucose), and YPD100 (100 g/L glucose) media, respectively. The initial OD600 values of cultures with SD50, YPD50, and YPD100 were 0.2, 0.2, and 0.5, respectively. (a–b) Time courses of (a) (*R*)-citramalate production and glucose concentration and (b) byproduct production in culture. (For interpretation of the references to color in this figure legend, the reader is referred to the Web version of this article.)

Batch fermentation was then conducted in flasks to evaluate the culture characteristics of ICM-dYAT2-comp. Baffled flasks (200 mL) were used to shake the ICM-dYAT2-comp culture in 40 mL SD50 medium containing 50 g/L glucose, 40 mL YPD50 medium, or 40 mL YPD100 medium containing 100 g/L glucose. In fermentations with SD50 and YPD50 media, ICM-dYAT2-comp completely consumed the glucose 72 h after the start of fermentation, producing approximately 13 mM (*R*)-citramalate (Fig. 8a). Interestingly, there was no difference in (*R*)-citramalate production or yield between SD50 and YPD50. Furthermore, in fermentation with YPD100, ICM-dYAT2-comp consumed approximately 75% of the glucose in the medium and produced 16.5 mM (2.5 g/L) (*R*)-citramalate for 96 h. This value is the highest reported for (*R*)-citramalate production by yeast to date. ICM-dYAT2-comp has a complete ethanol synthesis pathway, which likely explains why it produced ethanol 40-fold more than (*R*)-citramalate at 96 h (Fig. 8b). As most of the glucose-derived carbon is used in the synthesis of byproducts, such as ethanol and glycerol, in ICM-dYAT2-comp, suppressing their production is a future challenge. Table 1 summarizes the production of (*R*)-citramalate by recombinant *E. coli* and yeast. Although fed-batch fermentation with recombinant *E. coli* has been reported to produce up to 82 g/L (*R*)-citramalate, recombinant yeasts have only achieved up to 2.5 g/L, suggesting the need for further pathway engineering, including the suppression of ethanol production, to increase the production and yield of (*R*)-citramalate.

Chromosomal integration of genes for the overproduction of (*R*)-citramalate and evaluation of its production in the resulting strains. (a) Illustration of gene integration into yeast chromosome X. Five DNA fragments containing *cimA3.7*, *SpMAE1*, *An_xfpk*, *Ck_pta*, and *HIS3* were simultaneously integrated into a single site on yeast chromosome X using the CRISPR-Cas system, and the recombinant strains selected on SD medium lacking histidine. (b) Comparison of (*R*)-citramalate production among strains constructed in this study. All data are presented as averages of three independent experiments. Error bars represent standard deviations. Asterisks indicate significant differences as determined by Student's t-test (**P < 0.01).

## 4 Discussion

One reason why reports on (*R*)-citramalate production by recombinant microorganisms have been almost exclusively limited to prokaryotes may be the ease of intracellular acetyl-CoA utilization due to the absence of organelles. In *S. cerevisiae*, the presence of organelles makes it difficult to utilize cytosolic acetyl-CoA efficiently for the synthesis of target compounds. As mitochondria have higher concentrations of acetyl-CoA than the cytosol does (Weinert et al., 2014), they are often used as sites for the production of acetyl-CoA-derived compounds. However, the construction of pathways in mitochondria sometimes leads to the accumulation of metabolites, causing growth inhibition (Lv et al., 2016; Yuan and Ching, 2016). Efficient product secretion requires the synthesis of target compounds in the cytosol, and the establishment of a method to supply cytosolic acetyl-CoA is urgently needed for metabolic engineering of *S. cerevisiae*.

We found that either Δ*POR1* or Δ*YAT2* slightly increased (*R*)-citramalate production. Cytosolic acetyl-CoA is transported into the mitochondria in the form of acetyl-carnitine. This process, known as the carnitine shuttle, involves YAT1 and YAT2 converting cytosolic acetyl-CoA to acetyl-carnitine at the mitochondrial outer membrane and cytosol, respectively, while CRC1 transports acetyl-carnitine into the mitochondria at the inner membrane (Franken et al., 2008). Thus, our result that Δ*YAT2* increased (*R*)-citramalate production suggests that inhibiting acetyl-carnitine production in the cytosol is an effective method for increasing cytosolic acetyl-CoA accumulation.

MPC1, MPC2, and MPC3 are specialized transporters for pyruvate (Bricker et al., 2012; Herzig et al., 2012), and deletion of these genes increased the extracellular pyruvate concentration, whereas the production of (*R*)-citramalate was reduced, suggesting that the amount of cytosolic pyruvate was sufficiently high relative to the amount of cytosolic acetyl-CoA. POR1 and POR2 are mitochondrial porins (Di Rosa et al., 2021), and unlike MPC, which is specific for pyruvate transport, Δ*POR1* may alter the transport of various other small molecules into and out of mitochondria. POR1 is responsible for the uptake of external NADH into the mitochondrial inner membrane space, and Δ*POR1* may suppress oxidation of cytosolic NADH (Kmita and Budzińska, 2000; Avéret et al., 2002). In the current study, it is speculated that NADH oxidation is compensated for by metabolic reactions in the cytosol, and in fact, the Δ*POR1* disruption strain, ECM-dPOR1, showed increased ethanol synthesis compared to that of the other gene-knockout strains (Supplementary Fig. 2). This suggests that suppression of ethanol synthesis may lead to further NAD+/NADH imbalance and cause severe growth inhibition; therefore, metabolic manipulations for redox balancing are highly recommended when using the Δ*POR1* strain as a host strain. Cardenas and Da Silva (2016) found that single or multiple deletions of four genes (*MPC2*, *PDA1*, *POR2*, and *YAT2*) increased TAL production from cytosolic acetyl-CoA. *MPC2* and *POR2* are genes that did not cause an increase in (*R*)-citramalate production in this study. Thus, although different synthetic pathways may have different genes that can be deleted for cytosolic acetyl-CoA-derived compounds, Δ*YAT2* may be useful as a common means of inhibiting the first step of the carnitine shuttle, the formation of acetyl-carnitine. MPC1, MPC2, and MPC3 are subunits of the MPC present in the mitochondrial inner membrane, and MPCs mediate pyruvate uptake via MPC1 and MPC2 during fermentative growth and through MPC1 and MPC3 during respiratory growth (Bender et al., 2015). Therefore, the MPC genes that should be deleted may differ depending on culture conditions.

To improve cytosolic acetyl-CoA levels in *S. cerevisiae*, researchers have often constructed acetyl-CoA supply pathways derived from heterologous organisms (van Rossum et al., 2016). For example, pathways that convert acetaldehyde to acetyl-CoA by acetylating ALD (A-ALD) from *E. coli*, *Pseudomonas* sp., etc., the PDH complex from *E. coli* or *Enterococcus faecalis*, and the direct synthesis of acetyl-CoA from pyruvate via the citrate-oxaloacetate shuttle, which is transported from the mitochondria to the cytosol. Citric acid transported from the mitochondria to the cytosol is converted to acetyl-CoA via the cytosolic ATP-citrate lyase of *Arabidopsis thaliana* and *A*. *nidulans*, among others (van Rossum et al., 2016). As most acetyl-CoA supply pathways include reactions that require cofactors, such as NAD(P), it is important to design and construct a pathway that does not cause cofactor imbalance in cells.

The PK/PTA pathway can supply three molecules of acetyl-CoA from one molecule of glucose without carbon loss due to CO_2_ (van Rossum et al., 2016), but PK has two different activities, EC 4.1.2.9 and EC 4.1.2.22, the values and ratios of which vary with origin (Bergman et al., 2016). PTA is an enzyme that catalyzes a reversible reaction between acetyl-CoA and acetyl-phosphate. For example, PTA from *E. coli* has been found to have an approximately 20-fold higher affinity for acetyl-CoA than for acetyl-phosphate (Campos-Bermudez et al., 2010). In this study, several PK-PTA combinations were investigated for appropriate pathway construction. Interestingly, *in vitro* assays showed that the activity of the *A. nidulans* PK (0.02 and 0.06 U/mg for X5P and F6P, respectively) was significantly lower than that of the *L. mesenteroides* PK (1.05 and 0.19 U/mg for X5P and F6P, respectively) (Bergman et al., 2016). However, we found that the *A. nidulans* PK was the most suitable for (*R*)-citramalate production. This is an example of the difficulty in evaluating enzymes in metabolic engineering because their *in vitro* and *in vivo* activities may differ. Only Bl_xfspk has activity on sedoheptulose-7-phosphate, in addition to X5P and F6P, and a pathway for acetyl-CoA supply was recently designed to take advantage of this enzyme by simplifying the non-oxidative glycolysis pathway (Bogorad et al., 2013). Although we did not find any advantages for Bl_xfspk in this study, the reported pathway may be used to further improve (*R*)-citramalate production.

The budding yeast endogenous glycerol-3-phosphate phosphatases, GPP1 and GPP2, are also known to convert acetyl-phosphate to acetate, and GPP1 is responsible for the major activity of this reaction in the cell (Bergman et al., 2016). We speculated that if acetyl-phosphate is immediately converted to acetyl-CoA by PTA, the suppression of acetate formation by deleting *GPP1* would have a positive effect on (*R*)-citramalate production, but in fact, Δ*GPP1* had no effect. This indicates that acetyl-phosphate accumulates in the cytosol due to insufficient PTA activity, and that a more active PTA should be identified or artificially produced and combined with Δ*GPP1*.

Although dicarboxylate transporters have been identified in *S. cerevisiae*, such as the mitochondrial citrate transporter, CTP1, there are no known potent endogenous transporters suitable for organic acid production. In contrast, the heterologous expression of *SpMAE1* has been reported to significantly increase the extracellular concentrations of malate, fumarate, and succinate in *S. cerevisiae* (Darbani et al., 2019). We found that SpMAE1 was also effective in the efflux of (*R*)-citramalate, a 5-carbon dicarboxylic acid, indicating that SpMAE1 may be a means of improving the production of various dicarboxylic acids. The growth inhibition observed when *SpMAE1* was expressed in *2μ* plasmids appears to be improved in strains expressing it from chromosomes (Fig. 8 and Supplementary Fig. 1a), suggesting that proper expression of the gene may have contributed to the improved growth.

The ICM-dYAT2-comp strain was constructed with one copy each of *cimA3.7*, *An_xfpk*, *Ck_pta*, and *SpMAE1* integrated into the chromosome, and the proper copy number of these genes remains to be determined. In addition to further optimizing gene expression, the byproducts of ethanol and glycerol production must be suppressed to increase (*R*)-citramalate production. In fact, the final strain constructed, ICM-dYAT2-comp, produced 158 mM (7.3 g/L) ethanol and 16 mM (1.5 g/L) glycerol at 96 h of incubation in test tube culture (Supplementary Fig. 3). In *S. cerevisiae*, the synthesis of ethanol and glycerol plays a role in cytosolic NADH oxidation (Bakker et al., 2001). For example, the knockout of genes encoding acetaldehyde/alcohol dehydrogenase results in a significant reduction in growth due to an imbalance in redox cofactors and the accumulation of acetaldehyde, which subsequently leads to a decline in the productivity of target compounds (Song et al., 2016). The synthesis of (*R*)-citramalate does not oxidize excess NADH generated by deleting genes involved in ethanol or glycerol synthesis. Therefore, some manipulations are necessary to consume the toxic metabolic intermediate (Song et al., 2016) and maintain intracellular redox balance (Yamada et al., 2017) to achieve the high productivity of (*R*)-citramalate with lower byproduct levels.

(*R*)-citramalate is an important compound not only as a precursor of MMA, but also as a metabolic intermediate in higher alcohol synthesis in microbial metabolic engineering (Shi et al., 2016; Chen et al., 2017). In other words, (*R*)-citramalate has the potential to be an important intermediate for the synthesis of medium- and long-chain primary alcohols, which are industrially important compounds for biofuel production, in which the establishment of a high (*R*)-citramalate production pathway in microorganisms is highly significant. Currently, 1-butanol, 1-pentanol, 1-hexanol, and 1-octanol production has been reported in engineered *E. coli* strains (Zhang et al., 2008; Dekishima et al., 2011; Machado et al., 2012; Chen et al., 2017), whereas in *S. cerevisiae* the synthesis of linear alcohols with more than five carbons has not been achieved (Shi et al., 2016; Nishimura et al., 2018). Higher alcohol synthesis in yeast remains a challenge because the carbon chain elongation reaction of the intermediate in this pathway requires acetyl-CoA.

Although (*R*)-citramalate production in yeast is much lower than that in *E. coli*, there is still potential for further enhancement of production levels. When the pH of culture decreases below the pKa of organic acid metabolites such as acetic acid and (*R*)-citramalate, undissociated organic acids that exist extracellularly readily cross the cell membrane and cause growth inhibition (Mira et al., 2010). The production of engineered *E. coli* listed in Table 1 was obtained by adjusting the pH of the culture to 7.0 to avoid such acid stress. However, the recombinant yeasts constructed in this study did not require adjustment of culture pH. By closing the production gap with *E. coli* through further metabolic pathway optimization and achieving low-pH organic acid production that has low economical and environmental impact (Bhagwat et al., 2021), it would be advantageous to realize sustainable bioproduction. We hope that our findings on the metabolic engineering of a heterologous pathway in yeast to increase cytosolic acetyl-CoA levels will provide useful insights not only into (*R*)-citramalate production, but also into achieving higher alcohol synthesis in yeast in the future.

## 5 Conclusions

In this study, we reported, for the first time, the heterologous production of (*R*)-citramalate by engineered *S. cerevisiae*. To improve the supply of acetyl-CoA in the cytosol, which is required for (*R*)-citramalate synthesis, we inhibited cytosolic pyruvate and acetyl-CoA transport to the mitochondria, established a PK/PTA pathway, and heterologously expressed relevant transporters. All our attempts had a positive effect on increasing (*R*)-citramalate production, and combining them resulted in a large synergistic effect. The strain constructed in this study not only serves as a base strain for further optimization to construct (*R*)-citramalate high-producing yeast, but also provides valuable insights into the highly efficient production of useful compounds from cytosolic acetyl-CoA, a challenge in the metabolic engineering of budding yeast.

## Funding

This work was also supported by the 10.13039/501100002241Japan Science and Technology Agency GteX Program (Grant number JPMJGX23B0).

## CRediT authorship contribution statement

**Ryosuke Mitsui:** Writing – original draft, Supervision, Investigation, Formal analysis, Data curation, Conceptualization. **Akihiko Kondo:** Writing – review & editing, Resources. **Tomokazu Shirai:** Writing – review & editing, Funding acquisition.

## Declaration of competing interest

The authors declare that they have no known competing financial interests or personal relationships that could have appeared to influence the work reported in this paper

## Data Availability

No data was used for the research described in the article.
