# Supplementary material for: Production of (R)-citramalate by engineered Saccharomyces cerevisiae
Source: Metab Eng Commun. 2024 Aug 10;19:e00247. doi: 10.1016/j.mec.2024.e00247 (PMC11379666; doi:10.1016/j.mec.2024.e00247)
Supplement: Multimedia component 1 [file mmc1.docx]

**Supplementary data**

**Production of (*R*)-citramalate by engineered *Saccharomyces cerevisiae***

Ryosuke Mitsui ^a^, Akihiko Kondo ^a, b^, Tomokazu Shirai ^a^,*

^a^ Center for Sustainable Resource Science, RIKEN, 1-7-22, Suehiro-cho, Tsurumi-ku, Yokohama, Kanagawa, 230-0045, Japan

^b^ Graduate School of Science, Technology and Innovation, Kobe University, 1-1 Rokkodai, Nada, Kobe 657-8501, Japan

* Corresponding author. E-mail address: tomokazu.shirai@riken.jp

**S1. Plasmid construction**

*S1.1. Plasmids for cimA3.7, phosphoketolase genes, phosphotransacetylase genes, and SpMAE1 expression*

PCR was performed using KOD One (TOYOBO, Osaka, Japan) and the primer pairs listed in Supplementary Table 1. Eight heterologous genes, *cimA3.7*, *An_xfpk*, *Bb_xfpk*, *Bl_xfspk*, *Lm_xfpk*, *Bs_pta*, *Ck_pta*, and *SpMAE1*, were codon-optimized for *S. cerevisiae* and synthesized (Thermo Fisher Scientific, USA) (Supplementary Table 2). Each DNA fragment was assembled with the respective plasmid using NEBuilder HiFi DNA Assembly (New England Biolabs, Ipswich, MA, USA).

*PRM9* terminator (t*PRM9*) was amplified via PCR using *S. cerevisiae* YPH499 genome as the template and pPGK1-tPRM9_F and tPRM9-pGK4_R as the primer pair. Then, t*PRM9* was inserted into SacI/SalI site of pGK424. The resultant plasmid was named pEUsp.

p*PGK1*-t*PRM9* fragment was amplified via PCR using pEUsp as the template and pRS3-pPGK1_F and tPRM9-pRS3_R as the primer pair. p*PGK1*-t*PRM9* fragment was inserted into SacI/XhoI of pRS316. The resultant plasmids were named pCUsp.

The *cimA3.7* fragment was amplified via PCR using synthesized *cimA3.7* as the template and pPGK1-cimA3.7_F and cimA3.7-tPRM9_R as the primer pair, and then inserted into the SalI site of pEUsp and pCUsp. The resultant plasmids were named pEUsp-cimA3.7 and pCUsp-cimA3.7, respectively.

The region containing the ampicillin resistance gene and replication origin for *E. coli* was amplified by PCR using pRS316 as the template and AscI-pRS3_F and pRS3-AscI_R as the primer pair. Next, 500 bp up-/down-stream of PAM sequence in X-4 site were amplified by PCR using *S. cerevisiae* YPH499 genome as the template and pRS3-AscI-X-4_up_F and ChX-4_up_R, and X-4_down_F and ChX-4_down-AscI_R as the primer pairs, respectively. Then, these three fragments were assembled using NEBuilder HiFi DNA Assembly kit to construct pIntChX-4. After that, the p*PGK1*-*cimA3.7­­*-t*PRM9* fragment was amplified via PCR using synthesized pEUsp-cimA3.7 as the template and ChX-4_cimA3.7_F and tPRM9-ChX-4_R as the primer pair and then inserted into the EcoRI site of pIntChX-4. The resultant plasmids were named pIntChX-4-cimA3.7.

The *An_xfpk* fragment was amplified via PCR using synthesized *An_xfpk* as the template and pPGK1-An_xfpk_F and An_xfpk-tPGK1_R as the primer pair. The *Bb_xfpk* fragment was amplified via PCR using synthesized *Bb_xfpk* as the template and pPGK1-Bb_xfpk_F and Bb_xfpk-tPGK1_R as the primer pair. The *Bl_xfspk* fragment was amplified via PCR using synthesized *Bl_xfspk* as the template and pPGK1-Bl_xfspk_F and Bl_xfspk-tPGK1_R as the primer pair. The *Lm_xfpk* fragment was amplified via PCR using synthesized *Lm_xfpk* as the template and pPGK1-Lm_xfspk_F and Lm_xfspk-tPGK1_R as the primer pair. The *Bs_pta* fragment was amplified via PCR using synthesized *Bs_pta* as the template and pPGK1-Bs_pta_F and Bs_pta-tPGK1_R as the primer pair. The *Ck_pta* fragment was amplified via PCR using synthesized *Ck_pta* as the template and pPGK1- Ck_pta_F and Ck_pta-tPGK1_R as the primer pair. The *SpMAE1* fragment was amplified via PCR using synthesized *SpMAE1* as the template and pPGK1-SpMAE1_F and SpMAE1-tPGK1_R as the primer pair. pGK423 was digested with EcoRI and SalI to obtain pGK423/EcoRI/SalI. pGK424 was digested with EcoRI and NheI to obtain pGK424/EcoRI/NheI. The *Bs_pta*, *Ck_pta,* or *SpMAE1* fragment were assembled with pGK423/EcoRI/SalI to construct pGK423-Bs_pta, pGK423-Ck_pta, and pGK423-SpMAE1, respectively. Besides, the *An_xfpk*, *Bb_xfpk*, *Bl_xfspk*, or *Lm_xfpk* fragment were assembled with pGK424/EcoRI/NheI to construct pGK424-An_xfpk, pGK424-Bb_xfpk, pGK424-Bl_xfspk, and pGK424-Lm_xfpk, respectively.

| Supplementary Table 1 Primers used in this study | |
| --- | --- |
| Name | Sequence (5'-3') |
| pRS3-pPGK1_F | CTATAGGGCGAATTGAAAGATGCCGATTTGGG |
| tPRM9-pRS3_R | CCCCTCGAGGTCGACATTTTCAACATCGTATTTTC |
| pPGK1-cimA3.7_F | TTTTACAACAAATATAAAACATGATGGTCAGAATTTTCGAC |
| cimA3.7-tPRM9_R | TAGTGTCTCCCGTCTTCTGTTTAGACCAACTTACCAGTAAC |
| ChX-4_pPGK1_F | AAGCATTGTAGTAGAGTCAAAGATGCCGATTTGGG |
| tPRM9-ChX-4_R | GGAAACCTTTTCGTCCTATTTTCAACATCGTATTTTC |
| pPGK1-An_xfpk_F | TTTTTACAACAAATATAAAACATGCCAGGTGAAGTTATCG |
| An_xfpk-tPGK1_R | AATTTATTTCAGATCTTCAGTTGAAGGATGGCATG |
| pPGK1-Bb_xfpk_F | TTTTACAACAAATATAAAACATGACTAACCCAGTTATTGG |
| Bb_xfpk-tPGK1_R | ATTCAATTTATTTCAGATCTTCATTCATTATCACCAGCTG |
| pPGK1-Bl_xfspk_F | ACTTTTTACAACAAATATAAAACATGACCTCTCCAGTTATTG |
| Bl_xfspk-tPGK1_R | ATTCAATTTATTTCAGATCTTCATTCATTATCACCAGCTG |
| pPGK1-Lm_xfpk_F | TTACAACAAATATAAAACATGGCCGATTTCGACTC |
| Lm_xfpk-tPGK1_R | ATTCAATTTATTTCAGATCTTTACTTCAATGGTGACCAAG |
| pPGK1-Bs_pta_F | CAAATATAAAACGCTAGCATGGCCGATTTGTTCTC |
| Bs_pta-tPGK1_R | TTCAATTTATTTCAGATCTTCACAAAGCTTGAGCAGC |
| pPGK1-Ck_pta_F | CAAATATAAAACGCTAGCATGAAGCTGATGGAAAAC |
| Ck_pta-tPGK1_R | TTCAATTTATTTCAGATCTTTAACCTTGTGCTTGAGC |
| pPGK1-SpMAE1_F | CAAATATAAAACGCTAGCATGGGTGAACTCAAGG |
| SpMAE1-tPGK1_R | TTCAATTTATTTCAGATCTTTAAACGCTTTCATGTTCACTAC |
| pUCas9-pGAL1_F | AAATGCATGTATACGGATTAGAAGCCGCCG |
| tADH1-pUCas9_R | ACCGTAGAATTCCCGGTAGAGGTGTGGTCAATAAG |
| tADH1-pUCas9_F | TACCGGGAATTCTACGGTTATCCACAGAATCAGGG |
| pUCas9-pGAL1_R | AATCCGTATACATGCATTTACTTATAATACAG |
| pgRNA-EcoRI-pGAL1_F | GGTCCTTTTCGAATTCCGGATTAGAAGCCGCC |
| tCYC1-EcoRI-pgRNA_R | CCAGCAATTTTTACGGTTCGAATTCGCAAATTAAAGCCTTCG |
| pgRNA-ori_F | AACCGTAAAAATTGCTGGCGTTTTTCCATAGGCTCG |
| pgRNA-EcoRI_R | GAATTCGAAAAGGACCCAGGTGGCAC |
| ChX-4_up_F | TTTCTCTCCTTATCATCAAAATCTACAACCATGTAC |
| ChX-4_up_R | TTTTCGTCCTGAATTCCTCGAGGACTCTACTACAATGC |
| ChX-4_pPGK1_F2 | GCATTGTAGTAGAGTCAGACGCGAATTTTTCGAAG |
| ChX-4_pPGK1_R2 | ATTCGCGTCTGACTCTACTACAATGCTTTTTC |
| pPGK1-cimA3.7_F2 | AACAAATATAAAACAATGATGGTCAGAATTTTCGACAC |
| pPGK1-cimA3.7_R2 | TTCTGACCATCATTGTTTTATATTTGTTGTAAAAAGTAG |
| cimA3.7-tPRM9_F | GGTCTAAACAGAAGACGGGAGACACTAG |
| tPRM9-pTEF1_50_R | GAATATAGCGAAGATACCGATGAAGGATCCAATAACTCAAGTACGAACGG |
| tPRM9-pTEF1_50_F | CCGTTCGTACTTGAGTTATTGGATCCTTCATCGGTATCTTCGCTATATTC |
| pPGK1-SpMAE1_R | AGTTCACCCATTTTGTAATTAAAACTTAGATTAGATTGC |
| pPGK1-SpMAE1_F | AATTACAAAATGGGTGAACTCAAGGAAATCTTG |
| SpMAE1-tDIT1_R | CGCTCTTACTTTATTAAACGCTTTCATGTTCAC |
| SpMAE1-tDIT1_F | AAAGCGTTTAATAAAGTAAGAGCGCTACATTGG |
| tDIT1-pTPI1_50_R | GTTCCCTTATTCTTGGCTCATCCTTAAGTGAGTTCTATTCACGCAATCGG |
| tDIT1-pTPI1_50_F | CCGATTGCGTGAATAGAACTCACTTAAGGATGAGCCAAGAATAAGGGAAC |
| pTPI1-An_xfpk_R | CTTCACCTGGCATTTTTAGTTTATGTATGTGTTTTTTG |
| pTPI1-An_xfpk_F | ACATAAACTAAAAATGCCAGGTGAAGTTATCGAAAG |
| An_xfpk-tHXT7_R | GTTCGCAAATCAGTTGAAGGATGGCATG |
| An_xfpk-tHXT7_F | TCCTTCAACTGATTTGCGAACACTTTTATTAATTC |
| tHXT7-pTDH3_50_R | AAGGTGACAAGGTCCTCGAAAATAGCATAGATGCATTGTGAAAATTGAAG |
| tHXT7-pTDH3_50_F | CTTCAATTTTCACAATGCATCTATGCTATTTTCGAGGACCTTGTCACCTTG |
| pTDH3-Ck_pta_R | CAGCTTCATTTTGTTTGTTTATGTGTGTTTATTC |
| pTDH3-Ck_pta_F | CACATAAACAAACAAAATGAAGCTGATGGAAAAC |
| Ck_pta-tADH_R | AAATTCGCTTAACCTTGTGCTTGAGCTTG |
| Ck_pta-tADH_F | AAGCACAAGGTTAAGCGAATTTCTTATGATTTATG |
| tADH1-HIS3_50_R | TAGCGCTCACCAAGCTCTTAAAACGCGTAAAAAAAGCATGCACGTATACAC |
| tADH1-HIS3_50_F | TGTATACGTGCATGCTTTTTTTACGCGTTTTAAGAGCTTGGTGAGCGCTAG |
| tADH1-ChX-4_R | ACCTTTTCGTCCTCATATGATCCGTCGAGTTC |
| tADH1-ChX-4_F | GGATCATATGAGGACGAAAAGGTTTCCAGC |
| ChX-4_down_F | GAGTCCTCGAGGAATTCAGGACGAAAAGGTTTCCAG |
| ChX-4_down_R | GATCACCTCTTTGTTTAATCTTATGAATCCAAGGG |
| AscI-pRS3_F | GGCGCGCCTACGGTTATCCACAGAATC |
| pRS3-AscI_R | GGCGCGCCGAAAAGGACCCAGGTGG |
| pRS3-AscI-X-4_up_F | CCTTTTCGGCGCGCCTTTCTCTCCTTATCATC |
| tPRM9-pChX-4_R | TAACCGTAGGCGCGCCGATCCAATAACTCAAGTAC |
| pSpMAE1-pTEF1_F | TCCTTTTCGGCGCGCCCTTCATCGGTATCTTCGC |
| tDIT1-pSpMAE1_R | GATAACCGTAGGCGCGCCAAGTGAGTTCTATTCACG |
| pAn_xfpk-pTPI1_F | GTCCTTTTCGGCGCGCCAAGGATGAGCCAAGAATAAG |
| tHXT7-pAn_xfpk_R | ATAACCGTAGGCGCGCCCATAGATGCATTGTGAAAATTG |
| pCk_pta-pTDH3_F | GTCCTTTTCGGCGCGCCCTATTTTCGAGGACCTTG |
| tADH1-pCk_pta_R | ATAACCGTAGGCGCGCCCGTAAAAAAAGCATGCAC |
| pHIS3-ChX-4_down-HIS3_F | GTCCTTTTCGGCGCGCCGCGTTTTAAGAGCTTGGTG |
| ChX-4_down-AscI_R | AACCGTAGGCGCGCCGATCACCTCTTTGTTTAATC |
| donorDNA_up_CRC1-del_F | AAGCTTTATGCAGGGATCAGTTGGG |
| donorDNA_up_CRC1-del_R | AAAAATTTAATACTGACACGATGACGTTTGTGG |
| donorDNA_down_CRC1-del_F | CGTGTCAGTATTAAATTTTTGTTAGGTGGACC |
| donorDNA_down_CRC1-del_R | CATATCGGTTGATTATACTGTAATCAGCCCAC |
| donorDNA_up_GPP1-del_F | CTTTGTGTCTTTTAATTTTGACCATTTTGACC |
| donorDNA_up_GPP1-del_R | TAAAAGAAAATGCGATGGTTTGTATATTTGCTTTTG |
| donorDNA_down_GPP1-del_F | AACCATCGCATTTTCTTTTATTTTTTTGATAAAACTAC |
| donorDNA_down_GPP1-del_R | ACTTTTATAGTAAGATATAAAACAAAAACGAAGATTGAATCTTCC |
| donorDNA_up_MPC1-del_F | TTAAATATAATTTTATAACCCAGTTCTATATTGCTGGGTG |
| donorDNA_up_MPC1-del_R | GACTGCTCGTTTCTTTTTGATCACAGTGCAATAAAATCTATACG |
| donorDNA_down_MPC1-del_F | ATCAAAAAGAAACGAGCAGTCTAAGAACCTGAAGTAGGTG |
| donorDNA_down_MPC1-del_R | TAATCAGTAATACAAAGGAGATGAGAGGGAAATTGTC |
| donorDNA_up_MPC2-del_F | TATTGCGCGCATGACTAGGATGG |
| donorDNA_up_MPC2-del_R | AAATTCGGCGTTTTTCTTTTCTTTAATCTTTTTCTGTATAACTTC |
| donorDNA_down_MPC2-del_F | AAGAAAAACGCCGAATTTACAACTCGAAACTTTGC |
| donorDNA_down_MPC2-del_R | AAACTAGCCCTCTCAGAAAACACACC |
| donorDNA_up_MPC3-del_F | CCAATCGGCTATTAACGGCTTTACG |
| donorDNA_up_MPC3-del_R | GTTTACGTAATAAAAATATAGGTTTGTGTTTCTATGTGTC |
| donorDNA_down_MPC3-del_F | TATATTTTTATTACGTAAACGATAATATGTTCCTGAACTCG |
| donorDNA_down_MPC3-del_R | ATCCTGCTGCTAAAAAGGTCGTTG |
| donorDNA_up_POR1-del_F | GGGCGCTTGTCGCGTG |
| donorDNA_up_POR1-del_R | ATATATACGTTGTTGAGAGGTTGGTTTGATTTTTGC |
| donorDNA_down_POR1-del_F | CCTCTCAACAACGTATATATCTAATATATATATGTTCAC |
| donorDNA_down_POR1-del_R | CAATGTTCGAAACCAATCTGAAAATACCGAG |
| donorDNA_up_POR2-del_F | GCAAATGAGACGAATAACGGATTCCG |
| donorDNA_up_POR2-del_R | ATAAAATCTCCGTAGGTAATTTCTAACACTTTCCTC |
| donorDNA_down_POR2-del_F | GAAATTACCTACGGAGATTTTATTTTCTTATAAATTTTAC |
| donorDNA_down_POR2-del_R | AATTGGTGTGAAGCAGCGTATGTG |
| donorDNA_up_YAT1-del_F | CATAGTCCAAAGTAAAGGGGCAAGG |
| donorDNA_up_YAT1-del_R | CAGCATAAACTGTATACGGAAAGGAGGG |
| donorDNA_down_YAT1-del_F | TCCGTATACAGTTTATGCTGAGTTTTTGC |
| donorDNA_down_YAT1-del_R | TTTTTGGCAGTTTAATTTAGTTTGAGTGCTTC |
| donorDNA_up_YAT2-del_F | GAGGCAGCCCGTGTTGC |
| donorDNA_up_YAT2-del_R | AAGAGCGTTCGGTTGCTTAGTTTTATTTTAATTTTG |
| donorDNA_down_YAT2-del_F | CTAAGCAACCGAACGCTCTTTGTTTATCTATTTATTAC |
| donorDNA_down_YAT2-del_R | GTAAGTCACTCATGTCTACCTCTAATGCG |

| Supplementary Table 2 Sequence of synthetic genes |
| --- |
| *cimA3.7* (5’-3’) |
| ATGATGGTCAGAATTTTCGACACCACTTTGAGAGATGGTGAACAAACTCCAGGTGTTTCTTTGACTCCAAACGATAAGTTGGAAATCGCCAAAAAGTTGGATGAATTGGGTGTTGATGTTATCGAAGCTGGTTCTGCTGTTACTTCTAAAGGTGAAAGAGAAGGCATTAAGCTGATCACCAAAGAAGGTTTGAACGCCGAAATTTGCTCTTTCGTTAGAGCTTTGCCAGTTGATATTGATGCTGCTTTGGAATGTGATGTTGATTCCGTTCATTTGGTTGTTCCAACCTCTCCAATCCATATGAAGTACAAGTTGAGAAAGACCGAAGATGAAGTTTTGGTTACTGCTTTGAAGGCTGTTGAATACGCTAAAGAACAAGGTTTGATCGTTGAATTGTCTGCTGAAGATGCTACTAGATCCGATGTTAACTTCCTGATCAAGTTGTTCAACGAAGGTGAAAAGGTTGGTGCTGATAGAGTTTGTGTTTGTGATACTGTTGGTGTTTTGACCCCACAAAAATCTCAAGAGCTGTTCAAAAAGATCACCGAGAACGTTAATTTGCCAGTCTCTGTTCATTGCCATAACGATTTTGGTATGGCTACTGCTAATGCTTGCTCTGCTGTTTTAGGTGGTGCTGTTCAATGTCATGTTACTGTTAATGGTATTGGTGAGAGAGCTGGTAACGCTTCTTTGGAAGAAGTTGTTGCTGCTTCTAAAATCTTGTACGGTTACGATACCAAGATCAAGATGGAAAAGTTGTACGAAGTCTCCAGAATCGTGTCTAGATTGATGAAGTTGCCAGTTCCACCAAACAAAGCTATCGTTGGTGATAATGCTTTTGCTCATGAAGCCGGTATTCATGTTGATGGTTTGATTAAGAACACCGAAACCTACGAACCTATCAAGCCAGAAATGGTTGGTAACAGAAGAAGAATCATTTTGGGTAAGCACTCTGGTAGAAAGGCCCTAAAGTACAAATTGGATTTGATGGGCATCAACGTGTCCGATGAACAATTGAACAAAATCTACGAGAGGGTCAAAGAATTCGGTGACTTAGGTAAGTACATTTCCGATGCTGATTTGTTGGCTATCGTTAGAGAAGTTACTGGTAAGTTGGTCTAA |
| *An_xfpk* (5’-3’) |
| ATGCCAGGTGAAGTTATCGAAAGACCAAATCCAGCTCCAAAACCATCTCATGTTCCAGATTTGGTTGAGAAGTTGATTATCCCAGCTCAAAAGACCAAGTTGGAAAAGTCTGATTGTGATGCCTTGCATAAGTATAGAAGGGCTGCTGCTTATATTGCTGCTGGTCATTGGGGTACTTGTCCAGGTTTGATTTTGGTTTACTCCCACTTGAACTACCTGATCAAAAAGCAAAACTTGGACATGTTGTACGTTGTTGGTCCAGGTCATGGTGCTCCAGGTTTATTGGCTTCTTTGTGGTTGGAAGGTTCTTTGGGTAAATTCTATCCACAGTACACCAAAGACAAAGAAGGCTTGCATAACTTGATCTCCACTTTTTCTACATCTGCTGGTTTGCCATCTCATATTAACGCTGAAACTCCAGGTGCTATTCATGAAGGTGGTGAATTGGGTTATGCTTTGTCTGTTTCTTTTGGTGCCGTTATGGATAACCCAGATTTGATCGTTACTTGTGTTGTTGGTGATGGTGAAGCTGAAACTGGTCCAACTGCTACTTCTTGGCATGCTATTAAGTATATTGATCCAGCTGAATCCGGTGCTGTTTTGCCAATTTTACATGTTAACGGCTTCAAGATCTCCGAAAGAACTATTTTCGGTTGCATGGACAACAGAGAAATCGTTTGTTTGTTTACCGGTTACGGTTACCAAGTTAGAATCGTCGAAGATTTGGAAGATATCGATAACGACTTGCATTCTGCTATGTCTTGGGCTGTTGAAGAAATCAGAAACATTCAAAAGGCTGCCAGATCAGGTAAGCCAATTATGAAGCCACAATGGCCAATGATCGTTTTGAGAACTCCAAAAGGTTGGTCTGGTCCAAAAGAATTGCATGGTCAATTCATCGAAGGTTCCTTCCATTCTCATCAAGTTCCATTGCCAAACGCCAAAAAGGATGACGAAGAATTACAAGCCTTGCAGAAATGGTTGTCCTCTTACAAACCAGATGAGTTGTTTACTGAATCTGGTGATGTCATCGACGAGATCTTGTCTATTATTCCATCCGACGATAAGAAATTGGGTATGCGTCCAGAAGCTTACAAAACTCATTTGCCACCAGATTTGCCAGATTGGAGACAATTTTGTGTTAAGAAGGGTGATCAATTCTCCGCTATGAAGGCTATTGGTTCCTTCATTGATCAAGTGTTCGTTAAGAACCCACATACCGTTAGATTATTCTCCCCAGATGAATTGGAGTCCAACAAATTGTCTGCTGCTTTATCTCATACCGGTAGAAATTTCCAATGGGACGAATTCTCTAATGCCAAAGGTGGTAGAGTTATCGAGGTTTTGTCTGAACATTTGTGCCAAGGTTTCATGCAAGGTTATACTTTGACTGGTAGAACCGGTATTTTCCCATCTTACGAATCTTTCTTGGGTATCATCCATACCATGATGGTTCAATACGCTAAGTTTGCTAAGATGGCTAAAGAAACTGCTTGGCATCACGATGTTTCTTCCATTAACTACATTGAAACTTCCACCTGGGCTAGACAAGAACATAATGGTTTCTCTCATCAGAACCCATCTTTCATTGGTGCAGTTTTGAAATTGAAACCATACGCTGCTAGAGTTTACTTACCACCAGATGCTAATACTTTCTTGACTACCTTGCATCACTGCCTGAAGTCTAAGAACTACATCAATTTGATGGTCGGTTCTAAGCAACCTACTCCAGTTTATTTGTCTCCAGAAGAAGCTGAATCTCATTGCAGAGCTGGTGCTTCTATTTTCAAGTTCTGTTCTACTGACGGTGGTTTAAGACCAGATGTTGTTTTGGTTGGTATCGGTGTTGAAGTTATGTTCGAAGTTATTAAGGCTGCCGCCATTTTGAGAGAAAGATGTCCAGAATTGAGAGTCAGAGTTGTTAACGTTACCGACTTGTTCATCTTGGAAAATGAAGGTGCTCATCCACATGCCTTGAAACATGAAGCTTTTGATAACTTGTTCACCGAGGACAGATCCATCCATTTTAACTATCATGGTTACGTCAACGAGTTGCAGGGTTTGTTGTTTGGTAGACCAAGATTAGATAGAGCCACCATCAAAGGTTACAAAGAAGAAGGTTCTACTACTACCCCATTCGATATGATGTTGGTTAACGAAGTTTCCAGATACCATGTTGCTAAAGCTGCTGTTACTGGTGGTGCTAGATTCAACGAAAAAGTTAAGTTGAGGCACCAAGAATTGTGCTCCGAATTCGATCATAACATTGCCGAAACCAGAAAGTACATCATGAACAACCATCAAGATCCAGAGGATACTTACAACATGCCATCCTTCAACTGA |
| *Bb_xfpk* (5’-3’) |
| ATGACTAACCCAGTTATTGGTACTCCATGGCAAAAATTGGATAGACCAGTTTCTGAAGAGGCCATTGAAGGTATGGATAAGTATTGGAGAGTTACCAACTACATGTCCATCGGTCAAATCTACTTGAGATCTAACCCATTGATGAAGGAACCATTCACTAGAGATGATGTCAAGCACAGATTGGTTGGTCATTGGGGTACTACACCAGGTTTGAATTTCTTGTTGGCCCATATCAACAGATTGATCGCTGATCATCAACAGAACACCGTTTTCATTATGGGTCCAGGTCATGGTGGTCCAGCTGGTACTTCTCAATCTTATGTTGATGGTACTTACACCGAGTACTACCCAAACATTACAAAAGATGAAGCTGGCTTGCAGAAGTTCTTCAGACAATTTTCTTATCCAGGTGGTATCCCATCTCATTTTGCTCCAGAAACTCCAGGTTCTATTCATGAAGGTGGTGAATTGGGTTATGCTTTGTCTCATGCTTATGGTGCTGTTATGAACAACCCATCTTTGTTCGTTCCATGCATTATTGGTGATGGTGAAGCTGAAACAGGTCCATTGGCTACTGGTTGGCAATCTAACAAATTGGTTAACCCAAGAACCGATGGTATCGTTTTGCCAATCTTGCATTTGAACGGTTACAAGATTGCTAACCCAACCATTTTGGCCAGAATCTCTGATGAAGAATTGCACGATTTCTTCAGAGGTATGGGTTATCATCCATACGAATTTGTTGCTGGTTTCGACAACGAAGATCATATGTCCATTCATAGAAGATTCGCCGAGTTGTTCGAAACTATTTTCGACGAAATTTGCGATATTAAGGCTGCTGCTCAAACTGATGATATGACTAGACCATTTTACCCCATGTTGATTTTCAGAACTCCAAAAGGTTGGACCTGTCCAAAGTTTATCGATGGTAAAAAGACTGAAGGTTCTTGGAGAGCACATCAAGTTCCATTGGCATCTGCTAGAGATACTGAAGAACATTTCGAAGTCTTGAAAGGCTGGATGGAATCTTACAAACCTGAAGAGTTGTTTAACGCCGATGGTTCCATTAAGGATGATGTTACTGCTTTTATGCCAAAGGGTGAATTGAGAATTGGTGCTAATCCAAATGCTAACGGTGGTGTTATTAGGGAAGATTTGAAATTGCCAGAATTGGACCAATACGAAGTTACCGGTGTAAAAGAATATGGTCATGGTTGGGGTCAAGTTGAAGCTCCAAGAGCTTTGGGTGCTTACTGTAGAGATATTATCAAGAACAACCCCGACTCCTTCAGAATTTTTGGTCCAGACGAAACTGCTTCTAATAGATTGAATGCTACTTACGAAGTCACCGATAAGCAATGGGATAATGGTTATTTGTCCGGTTTGGTTGATGAACATATGGCTGTTACTGGTCAAGTTACCGAACAATTGTCAGAACATCAATGCGAAGGTTTCTTAGAAGCTTACTTGTTGACTGGTAGACATGGTATTTGGTCCTCTTACGAATCTTTCGTTCACGTTATCGACTCTATGTTGAATCAACACGCTAAGTGGTTGGAAGCTACCGTTAGAGAAATTCCTTGGAGAAAGCCAATCTCCTCTGTTAACTTGTTGGTTTCTTCACACGTTTGGAGACAAGATCATAACGGTTTCTCTCATCAAGATCCAGGTGTTACTTCCCTGTTGATTAACAAGACTTTCAACAACGATCACGTCACCAATATCTACTTTGCTACTGATGCTAACATGCTGTTGGCCATTTCTGAAAAGTGTTTCAAGTCTACCAACAAGATCAACGCTATTTTCGCTGGTAAACAACCAGCTCCAACTTGGGTTACTTTGGATGAAGCTAGAGCTGAATTGGAAGCTGGTGCTGCTGAATGGAAATGGGCTTCTAATGCTGAAAACAACGATGAAGTTCAAGTTGTTTTGGCTTCTGCTGGTGATGTTCCAACTCAAGAATTGATGGCTGCTTCTGATGCTTTAAACAAGATGGGTATCAAGTTCAAGGTCGTTAACGTTGTTGACTTGTTGAAGTTGCAGTCCAGAGAAAACAATGACGAAGCTTTGACTGACGAAGAATTCACTGAATTATTCACCGCTGATAAGCCAGTTTTGTTCGCTTATCATTCTTACGCCCAAGATGTCAGAGGTTTGATATACGATAGACCAAACCATGATAACTTCCACGTTGTCGGTTACAAAGAACAAGGTTCTACTACTACTCCATTCGATATGGTTAGAGTTAACGACATGGATAGATACGCTTTACAAGCTGCTGCTTTGAAGTTGATTGATGCAGATAAGTACGCCGATAAGATCGATGAATTGAACGCTTTTAGAAAGAAGGCTTTCCAATTCGCTGTTGATAACGGTTATGATATCCCAGAATTCACCGATTGGGTTTACCCAGATGTTAAGGTTGACGAAACTCAAATGTTGTCTGCTACAGCTGCAACAGCTGGTGATAATGAATGA |
| *Bl_xfspk* (5’-3’) |
| ATGACCTCTCCAGTTATTGGTACTCCATGGAAAAAGTTGAATGCCCCAGTTTCTGAAGAGGCTTTGGAAGGTGTTGATAAGTATTGGAGAGTTGCCAACTACTTGTCCATTGGTCAAATCTACTTGAGGTCTAACCCATTGATGAAGGAACCATTCACTAGGGAAGATGTTAAGCACAGATTGGTTGGTCATTGGGGTACTACACCAGGTTTGAATTTCTTGATTGGTCACATCAACAGATTCATTGCTGATCATGGTCAAAACACCGTTATTATCATGGGTCCAGGTCATGGTGGTCCAGCTGGTACTTCTCAATCTTATTTGGATGGTACTTACACCGAGACTTTCCCAAAGATTACAAAAGATGAAGCTGGCTTGCAGAAGTTCTTCAGACAATTTTCTTATCCAGGTGGTATCCCATCTCATTTTGCTCCAGAAACTCCAGGTTCTATTCATGAAGGTGGTGAATTGGGTTATGCTTTGTCTCATGCTTATGGTGCCATTATGGATAACCCATCTTTGTTTGTTCCAGCCATAGTTGGTGATGGTGAAGCTGAAACTGGTCCATTGGCTACTGGTTGGCAATCTAACAAATTGGTTAACCCAAGAACCGATGGTATCGTTTTGCCAATCTTGCATTTGAACGGTTACAAGATTGCTAACCCAACCATCTTGTCCAGAATCTCTGATGAAGAATTGCACGAATTCTTCCATGGTATGGGTTATGAACCATACGAATTTGTTGCTGGTTTCGATGATGAAGATCACATGTCCATTCATAGAAGATTCGCTGAATTGTGGGAAACCATTTGGGACGAAATTTGCGATATTAAGGCTACTGCTCAAACCGATAATGTTCACAGACCATTTTACCCCATGTTGATTTTCAGAACTCCAAAAGGTTGGACCTGTCCAAAGTATATCGATGGTAAAAAGACTGAAGGCTCTTGGAGATCTCATCAAGTTCCATTGGCATCTGCTAGAGATACTGAAGCTCATTTCGAAGTTTTGAAGAACTGGTTGGAAAGCTACAAACCTGAAGAGTTGTTTGATGCTAATGGTGCTGTTAAGGATGATGTTTTGGCTTTTATGCCAAAGGGTGAATTGAGAATTGGTGCTAATCCAAATGCAAACGGTGGTGTTATTAGAAACGATCTGAAGTTGCCAAACTTGGAAGATTACGAAGTCAAAGAAGTTGCCGAATACGGTCATGGTTGGGGTCAATTGGAAGCTACTAGAACTTTGGGTGCTTACACCAGAGATATTATCAAGAACAACCCAAGGGACTTCAGAATTTTTGGTCCAGACGAAACTGCTTCCAATAGATTGCAAGCTTCTTACGAAGTTACCAACAAACAATGGGATGCCGGTTACATTTCAGATGAAGTTGATGAACATATGCACGTGTCTGGTCAAGTTGTTGAACAATTGTCAGAACATCAGATGGAAGGTTTCTTGGAAGCTTATTTGTTGACAGGTAGACATGGTATCTGGTCCTCTTACGAATCTTTCGTTCATGTTATCGACTCCATGTTGAATCAACACGCTAAATGGTTAGAAGCCACCGTTAGAGAAATTCCTTGGAGAAAACCTATTGCCTCCATGAACTTGTTGGTTTCTTCACATGTTTGGAGACAAGATCACAACGGTTTTTCACATCAAGATCCAGGTGTTACCTCTGTCTTATTGAACAAGTGTTTCCACAACGATCACGTCATTGGTATCTACTTTGCTACTGATGCTAACATGTTGTTGGCTATTGCTGAAAAGTGTTACAAGTCCACCAACAAGATTAACGCTATTATTGCTGGTAAACAACCAGCTGCTACTTGGTTGACTTTGGATGAAGCTAGAGCTGAATTGGAAAAAGGTGCTGCTGCTTGGGATTGGGCTTCTACTGCTAAAAACAATGATGAAGCCGAAGTTGTTTTAGCTGCTGCTGGTGATGTTCCAACACAAGAAATTATGGCTGCTTCCGACAAGTTGAAAGAATTGGGTATTAAGTTCAAGGTTGTTAACGTTGCCGACTTGTTGTCTTTACAATCCGCTAAAGAAAACGACGAAGCTTTGACTGACGAAGAATTTGCTGATATCTTCACTGCTGATAAGCCAGTTTTGTTCGCTTATCATTCTTACGCCCATGATGTCAGAGGTTTGATATACGATAGACCAAACCACGATAACTTCAACGTTCATGGTTATGAAGAAGAAGGTTCTACTACTACCCCATACGATATGGTTAGAGTTAACAGAATCGACAGGTACGAATTGACTGCTGAAGCCTTGAGAATGATTGATGCAGATAAGTACGCTGACAAGATCGATGAATTGGAGAAGTTTAGAGATGAAGCCTTCCAATTCGCTGTTGATAATGGTTACGATCATCCAGATTACACCGATTGGGTTTACTCTGGTGTTAATACTGATAAGAAAGGTGCCGTTACTGCTACTGCTGCAACAGCTGGTGATAATGAATGA |
| *Lm_xfpk* (5’-3’) |
| ATGGCCGATTTCGACTCTAAAGAATACTTGGAATTGGTTGATAAGTGGTGGCGTGCTACTAATTACTTGTCTGCTGGTATGATCTTCTTGAAGTCTAACCCTTTGTTCTCCGTTACTAATACTCCAATCAAAGCCGAAGATGTTAAGGTTAAGCCAATTGGTCATTGGGGTACTATTTCTGGTCAAACTTTCTTGTACGCTCATGCCAACAGATTGATTAACAAGTACGGCCTGAATATGTTCTATGTTGGTGGTCCAGGTCATGGTGGTCAAGTTATGGTTACTAATGCTTATTTGGATGGTGCCTACACTGAAGATTACCCAGAAATTACCCAAGACATCGAAGGTATGTCTCACTTGTTTAAGAGATTCTCATTCCCAGGTGGTATCGGTTCTCATATGACTGCTCAAACTCCAGGTTCTTTACATGAAGGTGGTGAATTGGGTTACTCTTTGTCTCATGCTTTTGGTGCTGTTTTGGATAACCCAGATCAAGTTGCTTTTGCTGTTGTTGGTGATGGTGAAGCTGAAACTGGTCCATCTATGGCTTCATGGCATTCTATTAAGTTCTTGAACGCTAAGAATGATGGTGCCGTTTTGCCAGTTTTGGATTTGAATGGTTTCAAGATCTCTAACCCCACCATCTTCTCTAGAATGTCCGATGAAGAAATCACCAAGTTCTTTGAAGGTTTGGGTTACAGCCCAAGATTCATCGAAAACGATGATATTCATGATTACGCCACCTACCATCAATTGGCTGCTAACATTTTGGATCAAGCCATCGAAGATATCCAAGCCATTCAAAATGATGCCAGAGAAAACGGTAAATACCAAGATGGTGAAATTCCAGCTTGGCCAGTTATTATTGCTAGATTGCCAAAAGGTTGGGGTGGTCCAACTCATGATGCTTCTAACAATCCAATCGAAAACTCTTTCAGAGCCCATCAAGTTCCATTGCCATTGGAACAACATGATTTGGCTACTTTGCCAGAATTCGAAGATTGGATGAATTCCTACAAACCCGAAGAGTTGTTTAACGCTGATGGTTCCTTGAAGGATGAATTGAAAGCTATTGCTCCAAAGGGTGACAAAAGAATGTCTGCTAATCCAATTACTAATGGTGGTGCCGATAGATCCGATTTGAAATTGCCAAATTGGAGAGAATTCGCCAACGATATTAACGATGACACCAGAGGTAAAGAATTCGCTGATTCTAAGAGAAACATGGATATGGCTACCCTGTCTAACTATTTGGGTGCAGTTTCTCAATTGAACCCAACCAGATTCAGATTTTTCGGTCCAGACGAAACTATGTCTAATAGATTGTGGGGTTTGTTCAACGTTACTCCAAGACAATGGATGGAAGAGATCAAAGAACCACAAGATCAACTGTTGTCTCCAACCGGTAGAATTATTGACTCTCAATTGTCTGAACATCAAGCCGAAGGTTGGTTGGAAGGTTATACTTTGACTGGTAGAGTTGGTATTTTCGCCTCTTACGAATCTTTCTTGAGAGTTGTTGATACCATGGTTACCCAACATTTCAAGTGGTTGAGACATGCTTCAGAACAAGCTTGGAGAAATGATTACCCATCCTTGAACTTGATTGCTACTTCTACTGCTTTCCAACAAGATCATAACGGTTACACTCATCAAGATCCAGGTATGTTGACTCATTTGGCTGAAAAGAAGTCCAACTTCATCAGGGAATATTTGCCAGCTGATGGTAATTCTTTGTTGGCTGTCCAAGAAAGAGCCTTTTCTGAAAGACATAAGGTCAACTTGTTGATCGCTTCTAAGCAACCTAGACAACAATGGTTCACTGTTGAAGAAGCTGAAGTTTTGGCTAACGAAGGTTTGAAGATTATTGATTGGGCTTCTACAGCTCCATCCTCCGATGTTGATATTACTTTTGCTTCAGCTGGTACTGAGCCAACTATTGAAACTTTGGCTGCTTTGTGGTTGATTAACCAGGCTTTTCCTGATGTCAAGTTCAGATACGTTAATGTCGTCGAACTATTGAGGCTGCAAAAGAAATCTGAACCTAACATGAACGACGAGAGAGAATTATCTGCTGAAGAGTTCAACAAGTACTTCCAAGCTGATACCCCAGTTATTTTTGGTTTCCATGCTTACGAAAACCTGATCGAGTCATTTTTCTTCGAACGTAAATTCACTGGTGATGTTTACGTTCACGGTTATAGAGAGGATGGCGATATTACTACTACCTACGATATGAGAGTTTACTCCCACTTGGATAGATTCCATCAAGCTAAAGAAGCTGCCGAAATTTTGTCCGCTAATGGTAAAATTGATCAAGCTGCTGCTGATACCTTCATTGCTAAAATGGATGATACTTTGGCCAAGCACTTTCAAGTTACTAGAAACGAAGGTAGGGATATCGAAGAATTCACCGATTGGACTTGGTCACCATTGAAGTAA |
| *Bs_pta* (5’-3’) |
| ATGGCCGATTTGTTCTCTACCGTTCAAGAAAAAGTTGCTGGTAAGGATGTCAAGATCGTTTTTCCAGAAGGTTTGGACGAAAGAATTTTGGAAGCTGTTTCTAAATTGGCCGGTAACAAGGTTTTGAACCCAATCGTTATTGGTAACGAAAACGAAATTCAAGCCAAGGCCAAAGAATTGAACTTGACTTTAGGTGGCGTTAAGATCTACGATCCACATACTTATGAAGGCATGGAAGATTTGGTTCAAGCCTTCGTTGAAAGAAGAAAAGGTAAGGCTACTGAAGAACAAGCTAGAAAAGCTTTGTTGGACGAGAATTACTTCGGTACTATGTTGGTCTACAAAGGTTTGGCTGATGGTTTGGTTTCTGGTGCTGCTCATTCTACTGCTGATACTGTTAGACCAGCATTGCAAATCATCAAGACAAAAGAGGGTGTCAAAAAGACCTCCGGTGTTTTCATTATGGCTAGAGGTGAAGAACAGTATGTTTTCGCTGATTGCGCTATTAACATTGCCCCAGATTCTCAAGATTTGGCCGAAATTGCTATTGAATCTGCTAACACTGCTAAGATGTTCGACATTGAACCTAGAGTTGCTATGTTGTCATTCTCTACAAAAGGTTCTGCTAAGTCTGACGAAACTGAAAAGGTTGCTGATGCAGTTAAGATCGCTAAAGAAAAAGCTCCAGAATTGACCTTGGATGGTGAATTTCAATTTGATGCTGCTTTCGTTCCATCCGTTGCTGAAAAGAAAGCTCCTGATTCTGAAATCAAGGGTGATGCCAATGTTTTCGTCTTTCCATCTTTAGAAGCTGGTAACATCGGTTACAAGATTGCTCAAAGATTGGGTAACTTTGAAGCTGTTGGTCCAATATTGCAAGGTTTGAATATGCCAGTTAACGACTTGTCTAGAGGTTGTAATGCTGAAGATGTTTACAACTTGGCTTTGATTACTGCTGCTCAAGCTTTGTGA |
| *Ck_pta* (5’-3’) |
| ATGAAGCTGATGGAAAACATTTTCGGTTTGGCTAAGGCTGACAAGAAGAAAATCGTTTTGGCTGAAGGTGAAGAAGAGAGAAATATTAGAGCCTCCGAAGAGATTATCAGAGATGGTATTGCTGATATCATCTTGGTCGGTTCTGAATCCGTTATCAAAGAAAATGCTGCTAAGTTCGGTGTTAACTTGGCTGGTGTTGAAATAGTTGATCCAGAAACTTCTTCTAAGACTGCTGGTTACGCTAATGCCTTTTACGAAATCAGAAAGAACAAGGGTGTTACCTTGGAAAAGGCTGATAAGATAGTTAGGGATCCAATCTACTTCGCTACCATGATGGTTAAGTTGGGTGATGCTGATGGTTTGGTTTCTGGTGCTATTCATACAACCGGTGATTTGTTAAGACCAGGCTTGCAAATTGTCAAAACTGTTCCAGGTGCTTCCGTTGTTTCTTCTGTTTTCTTGATGTCTGTTCCAGATTGCGAATATGGTGAAGATGGTTTCTTGTTGTTTGCTGATTGTGCTGTTAACGTTTGTCCAACCGCTGAAGAATTATCCTCTATTGCTATTACTACTGCTGAAACCGCTAAGAACTTGTGCAAAATTGAACCTAGAGTTGCCATGTTGTCTTTCTCTACTATGGGTTCTGCTTCCCATGAATTGGTTGATAAGGTTACTAAGGCTACCAAGTTGGCTAAAGAAGCTAGACCAGATTTGGATATCGATGGTGAATTACAATTGGATGCCTCCTTGGTTAAGAAGGTTGCTGATTTGAAAGCTCCAGGTTCTAAAGTTGCTGGTAAGGCTAATGTTTTGATCTTCCCAGATATTCAAGCTGGTAACATCGGTTACAAGTTGGTTCAAAGATTCGCTAAAGCTGAAGCCATTGGTCCAATTTGTCAAGGTTTTGCTAAGCCAATCAACGACTTGTCTAGAGGTTGTTCTGTTGATGATATCGTTAAGGTTGTTGCCGTTACTGCTGTTCAAGCTCAAGCACAAGGTTAA |
| *SpMAE1* (5’-3’) |
| ATGGGTGAACTCAAGGAAATCTTGAAACAGAGGTATCATGAGTTGCTTGACTGGAATGTCAAAGCCCCTCATGTCCCTCTCAGTCAACGACTGAAGCATTTTACATGGTCTTGGTTTGCATGTACTATGGCAACTGGTGGTGTTGGTTTGATTATTGGTTCTTTCCCCTTTCGATTTTATGGTCTTAATACAATTGGCAAAATTGTTTATATTCTTCAAATCTTTTTGTTTTCTCTCTTTGGATCATGCATGCTTTTTCGCTTTATTAAATATCCTTCAACTATCAAGGATTCCTGGAACCATCATTTGGAAAAGCTTTTCATTGCTACTTGTCTTCTTTCAATATCCACGTTCATCGACATGCTTGCCATATACGCCTATCCTGATACCGGCGAGTGGATGGTGTGGGTCATTCGAATCCTTTATTACATTTACGTTGCAGTATCCTTTATATACTGCGTAATGGCTTTTTTTACAATTTTCAACAACCATGTATATACCATTGAAACCGCATCTCCTGCTTGGATTCTTCCTATTTTCCCTCCTATGATTTGTGGTGTCATTGCTGGCGCCGTCAATTCTACACAACCCGCTCATCAATTAAAAAATATGGTTATCTTTGGTATCCTCTTTCAAGGACTTGGTTTTTGGGTTTATCTTTTACTGTTTGCCGTCAATGTCTTACGGTTTTTTACTGTAGGCCTGGCAAAACCCCAAGATCGACCTGGTATGTTTATGTTTGTCGGTCCACCAGCTTTCTCAGGTTTGGCCTTAATTAATATTGCGCGTGGTGCTATGGGCAGTCGCCCTTATATTTTTGTTGGCGCCAACTCATCCGAGTATCTTGGTTTTGTTTCTACCTTTATGGCTATTTTTATTTGGGGTCTTGCTGCTTGGTGTTACTGTCTCGCCATGGTTAGCTTTTTAGCGGGCTTTTTCACTCGAGCCCCTCTCAAGTTTGCTTGTGGATGGTTTGCATTCATTTTCCCCAACGTGGGTTTTGTTAATTGTACCATTGAGATAGGTAAAATGATAGATTCCAAAGCTTTCCAAATGTTTGGACATATCATTGGGGTCATTCTTTGTATTCAGTGGATCCTCCTAATGTATTTAATGGTCCGTGCGTTTCTCGTCAATGATCTTTGCTATCCTGGCAAAGACGAAGATGCCCATCCTCCACCAAAACCAAATACAGGTGTCCTTAACCCTACCTTCCCACCTGAAAAAGCACCTGCATCTTTGGAAAAAGTCGATACACATGTCACATCTACTGGTGGTGAATCGGATCCTCCTAGTAGTGAACATGAAAGCGTTTAA |

*S1.2. Plasmids for gene deletion*

Gene deletion was basically performed according to the previous method (Okada et al., 2021). Gene deletion was achieved by completely removing the ORF of the target gene using CRISPR-Cas system. To construct a plasmid expressing Cas9 and gRNA, 20 nt of upstream sequences of PAM sequence in the target gene was determined from candidates shown in CRISPRdirect (https://crispr.dbcls.jp/). Single-stranded DNAs including 20 nt of sequence determined above were synthesized for each gene (Eurofins Genomics, Tokyo, Japan) (Supplementary Table 3) and inserted into BsaI site of pGAL1-Cas9-tADH1-pGAL1-2BsaI-sgRNAFE (empty)-HDV-tCYC1-CU using NEBridge Golden Gate Assembly Kit (BsaI-HF v2) (New England Biolabs). The resultant plasmid was named pCRISPR-Xdel (X: corresponding to target gene).

| Supplementary Table 3 DNA fragments for Golden Gate Assembly | | |
| --- | --- | --- |
| Target | Name | sequence (5'-3') |
| *CRC1* | fwd-oligo_CRC1-del | GGAGTTTGATCTGATGAGTCCGTGAGGACGAAACGAGTAAGCTCGTCATCAAATCATTTGTGGCCGG |
|  | rev-oligo_CRC1-del | AAACCCGGCCACAAATGATTTGATGACGAGCTTACTCGTTTCGTCCTCACGGACTCATCAGATCAAA |
| *GPP1* | fwd-oligo_GPP1-del | GGAGGAATAACTGATGAGTCCGTGAGGACGAAACGAGTAAGCTCGTCTTATTCACATCTCTCACGGT |
|  | rev-oligo_GPP1-del | AAACACCGTGAGAGATGTGAATAAGACGAGCTTACTCGTTTCGTCCTCACGGACTCATCAGTTATTC |
| *MPC1* | fwd-oligo_MPC1-del | GGAGAGCAAACTGATGAGTCCGTGAGGACGAAACGAGTAAGCTCGTCTTTGCTTTAGTTACCTATTC |
|  | rev-oligo_MPC1-del | AAACGAATAGGTAACTAAAGCAAAGACGAGCTTACTCGTTTCGTCCTCACGGACTCATCAGTTTGCT |
| *MPC2* | fwd-oligo_MPC2-del | GGAGGTAGGACTGATGAGTCCGTGAGGACGAAACGAGTAAGCTCGTCTCCTACTTTGAAATGGGGTC |
|  | rev-oligo_MPC2-del | AAACGACCCCATTTCAAAGTAGGAGACGAGCTTACTCGTTTCGTCCTCACGGACTCATCAGTCCTAC |
| *MPC3* | fwd-oligo_MPC3-del | GGAGGTGCCGCTGATGAGTCCGTGAGGACGAAACGAGTAAGCTCGTCCGGCACTGATTTGGACGCGT |
|  | rev-oligo_MPC3-del | AAACACGCGTCCAAATCAGTGCCGGACGAGCTTACTCGTTTCGTCCTCACGGACTCATCAGCGGCAC |
| *POR1* | fwd-oligo_POR1-del | GGAGTTCAGACTGATGAGTCCGTGAGGACGAAACGAGTAAGCTCGTCTCTGAACCTGTTCACAAGCT |
|  | rev-oligo_POR1-del | AAACAGCTTGTGAACAGGTTCAGAGACGAGCTTACTCGTTTCGTCCTCACGGACTCATCAGTCTGAA |
| *POR2* | fwd-oligo_POR2-del | GGAGGATGGGCTGATGAGTCCGTGAGGACGAAACGAGTAAGCTCGTCCCCATCCAAACTAGCGTAGA |
|  | rev-oligo_POR2-del | AAACTCTACGCTAGTTTGGATGGGGACGAGCTTACTCGTTTCGTCCTCACGGACTCATCAGCCCATC |
| *YAT1* | fwd-oligo_YAT1-del | GGAGTATTCTCTGATGAGTCCGTGAGGACGAAACGAGTAAGCTCGTCAGAATACGACGCACGGCTCG |
|  | rev-oligo_YAT1-del | AAACCGAGCCGTGCGTCGTATTCTGACGAGCTTACTCGTTTCGTCCTCACGGACTCATCAGAGAATA |
| *YAT2* | fwd-oligo_YAT2-del | GGAGCCTGAACTGATGAGTCCGTGAGGACGAAACGAGTAAGCTCGTCTTCAGGACGATGTCCTACCC |
|  | rev-oligo_YAT2-del | AAACGGGTAGGACATCGTCCTGAAGACGAGCTTACTCGTTTCGTCCTCACGGACTCATCAGTTCAGG |
| X-4 site | fwd-oligo_ChX-4 | GGAGTGAGCCCTGATGAGTCCGTGAGGACGAAACGAGTAAGCTCGTCGGCTCACTGAAAAACCGGGG |
|  | rev-oligo_ChX-4 | AAACCCCCGGTTTTTCAGTGAGCCGACGAGCTTACTCGTTTCGTCCTCACGGACTCATCAGGGCTCA |

*S1.3. Plasmids for multiple gene insertion*

For CRISPR-Cas system to cleave chromosome X-4 site, pUCas9 and pgRNA_ChX-4 were constructed as follows. The DNA fragment including pGAL1-Cas9-tADH1 was amplified via PCR using pGAL1-Cas9-tADH1-pGAL1-2BsaI-sgRNAFE (empty)-HDV-tCYC1-CU as the template and pUCas9-pGAL1_F and tADH1-pUCas9_R as the primer pair. Also, the backbone was amplified via PCR using pGAL1-Cas9-tADH1-pGAL1-2BsaI-sgRNAFE (empty)-HDV-tCYC1-CU as the template and tADH1-pUCas9_F and pUCas9-pGAL1_R as the primer pair. Then, these two fragments were assembled to construct pUCas9. Besides, the DNA fragment including pGAL1-2BsaI-sgRNAFE (empty)-HDV-tCYC1 was amplified via PCR using pGAL1-Cas9-tADH1-pGAL1-2BsaI-sgRNAFE (empty)-HDV-tCYC1-CU as the template and pgRNA-EcoRI-pGAL1_F and tCYC1-EcoRI-pgRNA_R as the primer pair. Also, the backbone was amplified via PCR using pGAL1-Cas9-tADH1-pGAL1-2BsaI-sgRNAFE (empty)-HDV-tCYC1-CU as the template and pgRNA-ori_F and pgRNA-EcoRI_R as the primer pair. Then, these two fragments were assembled to construct pgRNA. Then, fwd-oligo_ChX-4 and rev-oligo_ChX-4 were inserted into BsaI site using NEBridge Golden Gate Assembly Kit to construct pgRNA_ChX-4.

To prepare DNA fragments for chromosomal integration, five plasmids were constructed as follows. Each DNA fragment was amplified via PCR according to Supplementary Table 4. Next, PCR products from No. 1 to No. 4, PCR products from No. 5 to No. 7, PCR products from No. 8 to No. 10, PCR products from No. 11 to No. 13, PCR products from No. 14 and No. 15, were fused by overlap extension PCR using the corresponding primer pairs, respectively (No. 17-21). Then, each fused fragment was assembled with the PCR product No. 16 using NEBuilder HiFi DNA Assembly kit. The resultant plasmids were named pChX-4_up_cimA3.7, pSpMAE1, pAn_xfpk, pCk_pta, and pHIS3_ChX-4_down, respectively.

| Supplementary Table 4 List of PCR templates and primers for the construction of plasmids for gene insertion | | | |
| --- | --- | --- | --- |
| No. | Template | Forward primer | Reverse primer |
| 1 | YPH499 genome | ChX-4_up_F | ChX-4_pPGK1_R2 |
| 2 | YPH499 genome | ChX-4_pPGK1_F2 | pPGK1-cimA3.7_R2 |
| 3 | pEUsp-cimA3.7 | pPGK1-cimA3.7_F2 | cimA3.7-tPRM9_R |
| 4 | YPH499 genome | cimA3.7-tPRM9_F | tPRM9-pTEF1_50_R |
| 5 | YPH499 genome | tPRM9-pTEF1_50_F | pTEF1-SpMAE1_R |
| 6 | Synthetic SpMAE1 | pTEF1-SpMAE1_F | SpMAE1-tDIT1_R |
| 7 | YPH499 genome | SpMAE1-tDIT1_F | tDIT1-pTPI1_50_R |
| 8 | YPH499 genome | tDIT1-pTPI1_50_F | pTPI1-An_xfpk_R |
| 9 | Synthetic An_xfpk | pTPI1-An_xfpk_F | An_xfpk-tHXT7_R |
| 10 | YPH499 genome | An_xfpk-tHXT7_F | tHXT7-pTDH3_50_R |
| 11 | YPH499 genome | tHXT7-pTDH3_50_F | pTDH3-Ck_pta_R |
| 12 | Synthetic Ck_pta | pTDH3-Ck_pta_F | Ck_pta-tADH_R |
| 13 | YPH499 genome | Ck_pta-tADH_F | tADH1-HIS3_50_R |
| 14 | pRS313 | tADH1-HIS3_50_F | tADH1-ChX-4_R |
| 15 | YPH499 genome | tADH1-ChX-4_F | ChX-4_down_R |
| 16 | pRS316 | AscI-pRS3_F | pRS3-AscI_R |
| 17 | No. 1, 2, 3, 4 | pRS3-AscI-ChX-4_up_F | tPRM9-pChX-4_R |
| 18 | No. 5, 6, 7 | pSpMAE1-pTEF1_F | tDIT1-pSpMAE1_R |
| 19 | No. 8, 9, 10 | pCk_pta-pTDH3_F | tADH1-pCk_pta_R |
| 20 | No. 11, 12, 13 | pAn_xfpk-pTPI1_F | tHXT7-pAn_xfpk_R |
| 21 | No. 14, 15 | pHIS3-ChX-4_down-HIS3_F | ChX-4_down-AscI_R |

**S.2. Gene deletion and gene insertion**

*S2.1. Gene deletion*

Donor DNA used to replace ORF of the target gene was prepared via PCR using *S. cerevisiae* YPH499 genome as the template and appropriate primer pairs shown in Supplementary Table 1. For example, for *CRC1* deletion, 300 bp of upstream of the start codon was amplified via PCR using donorDNA_up_CRC1-del_F and donorDNA_up_CRC1-del_R as the primer pair. Also, the downstream of the end codon was amplified via PCR using donorDNA_down_CRC1-del_F and donorDNA_down_CRC1-del_R as the primer pair. And then, these two DNA fragments were fused by overlap extension PCR using donorDNA_up_CRC1-del_F and donorDNA_down_CRC1-del_R as the primer pair to construct donor DNA for CRC1 deletion. The donor DNA was concentrated to 10 μL of sterile water by ethanol precipitation, and then used for yeast transformation with 1 μL of plasmid pCRISPR-Xdel (X: corresponding to the gene to be deleted). The gene knockout strains were selected via PCR from the colonies formed on SG-U plate medium.

*S.2.2. Gene insertion*

For insertion of only the *cimA3.7* expression cassette into the chromosome X-4 site (Supplementary Table 5), 1.5 μg of pIntChX-4_cimA3.7 was digested with AscI (37ºC for 2 h) and concentrated to 10 μL of sterile water by ethanol precipitation, and then used for transformation. For insertion of multiple gene expression cassettes, five DNA fragments obtained via PCR using five plasmids pChX-4_up_cimA3.7, pSpMAE1, pAn_xfpk, pCk_pta, and pHIS3_ChX-4_down as templates, respectively, with the corresponding primers (Supplementary Table 1) were mixed at 0.3 pmol each in a single tube. This solution was ethanol precipitated and the precipitate was dissolved in 10 μL of sterile water and used for transformation. A yeast strain cultured in YPD for 1-3 days and incubated in YPG media was transformed with 1.5 μg of pUCas9 and 2.0 μg of pgRNA_ChX-4, along with the ethanol-precipitated DNA solution above. After that, transformants were spread on SG lacking uracil and histidine medium and incubated at 30ºC.

| Supplementary Table 5 X-4 site on chromosome X |
| --- |
| Sequence of chromosome X 236101…237400 (5’-3’) |
| AAAGGGACGAATCCTCCATATTAAGCTCAGTGAGTATCTTGTCATGGCAAACGTCGAGCATAGAATTTCTCTCCTTATCATCAAAATCTACAACCATGTACTCAAGCTCGTCTCCCCAAAAAAGAGGGTCATTGTCTCTTTTACCAGCAGCTTGGAAAATATACAACAACTGCTCGATACCTTCATCCCTTATGTGTTCATTGTACGTCCTAGACTCAAACCACTGCAAAGGCGTGCCCAAAGCTAAGAGTCCCATTTTATTCTTCTATATGTATATTTTCGATACTCTAAACCACCCTACAATGTAGCCCTATACTAAATCTGCTCAATTTTCAGCTTCTACAAGTGACTCGAGACCACGTGGAAAGATCCAACTACTCCAGCACAACGATTCAATATAATCGATTGCTCCACTCATAAGAGGCAAGAACAAGCTTCAACTTTTGGTAAGCCGCCGTTTATAAACAGGGAAGATGTCCTTTGTCAAGGGAGGCACAGAGCATGGCCAATTTGGCAAATTGCAGGTTTTTCTGAGTGAAAAATGAAAAAGCATTGTAGTAGAGTCGGCTCACTGAAAAACCGGGGAGGACGAAAAGGTTTCCAGCCACAGTTGTAGTCACGTGCGCGCCATGCTGACTAATGGCAGCCGTCGTTGGGCAGAAGAGAATTAGTATGGTACAGGATACGCTAATTGCGCTCCAACTACCAAGGTTGTTGAGGGAACACTGGGGCAATAGGCTGTCGCCATTCAAGAGCAGCAACAGGCATGGGAAGATTCGCTTTTTTTTTTTGAATTACAATAGTATGTCTGATGTCTGCAAGAAGTAACAGGCGTGTGCACAAGAATACGTGTGTGTGCGTAAGCGTATGCACTGGTGGCATAACTTATCTAAGAAGTATATATCACTGACATAGAAATGTAGATATACAGGTATTTTTCTCGATAATCGATAAAAATCTCGTCGCGCTGAACCAAACTTGGTGGTTACGGAGAGTTTTTCTCTCATCATTACTGTCTTTCGCATTGATTTCCCCTTTGACCGATAAAATCCCTTGGATTCATAAGATTAAACAAAGAGGTGATCAAAGAGAACCCTGTGAAAGTTTATGTTTATAACCGGGCATAAAGTGAACTAGACACTTTCAAGAAGCCAACCAAAGCATGAGTAACGAAGCTTACCAGCATGATCATACCGTAAATCCTCACCAGAAGATAGTTGTGAACAGCTACGATTGGTTACAGTTCCGTGATGAGCAAGATCACTGTAAAAGTAAGAACCCGATAACGCACGCTTCTCCA |

Homologous region for gene insertion

Target sequence for CRISPR-Cas

*S. 2.3. Plasmid removal*

A plasmid in yeast was removed by cultivating the yeast in YPD medium at 30ºC with agitation (180 rpm) for 2-3 days. Plasmid removal was confirmed by cultivating the yeast on SD plate media lacking uracil , YPD plate media, or SD plate media containing 1% (w/v) 5-fluoroorotic acid (5-FOA) (FUJIFILM Wako, Osaka, Japan).

**S.3. Supplementary figures**


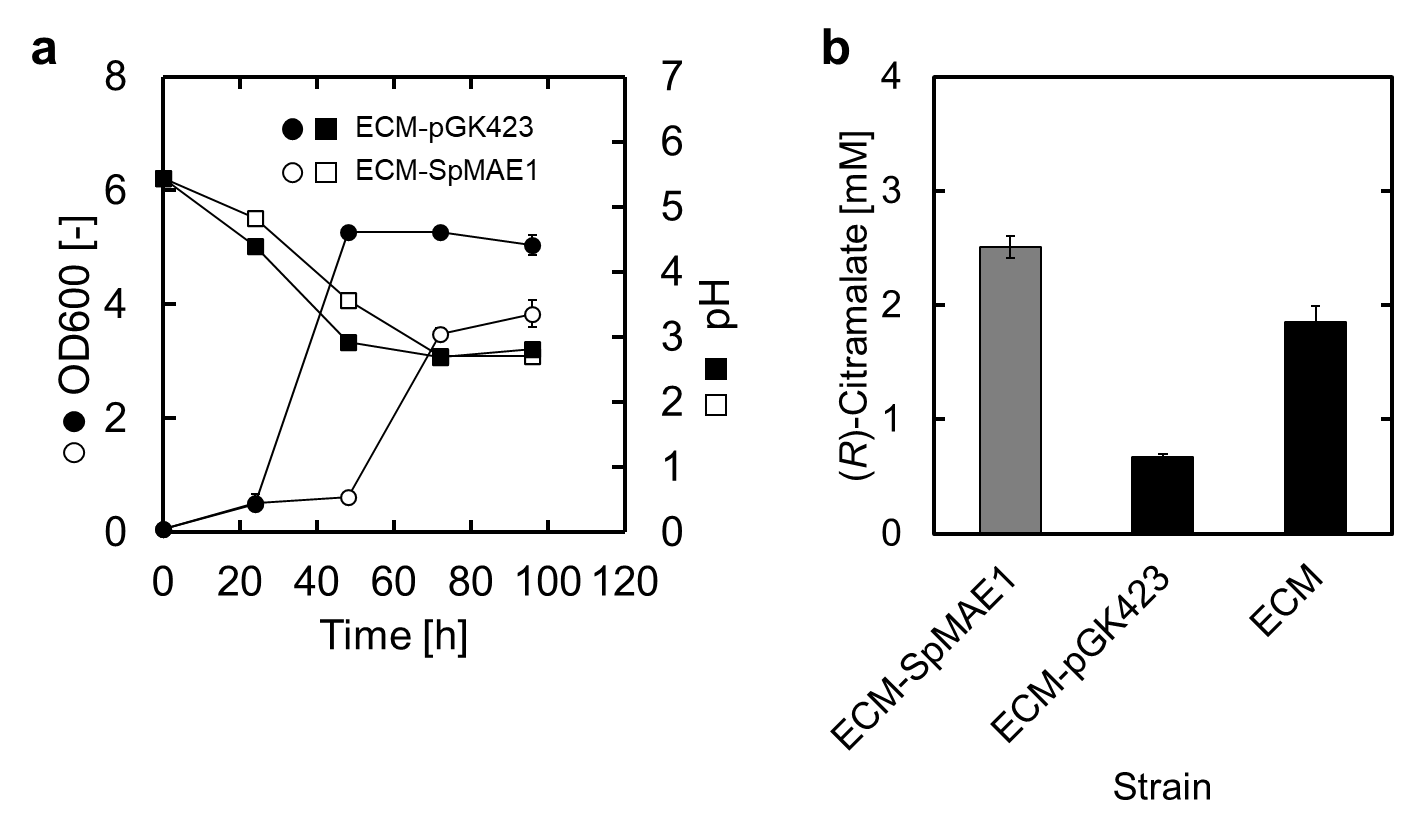


**Supplementary Fig. 1. Growth and (*R*)-citramalate concentration of a strain expressing a heterologous malate transporter *SpMAE1***

(a) Time course of OD600 and culture pH of strains expressing *SpMAE1* (ECM-SpMAE1) or not (ECM-pGK423). (b) Comparison of (*R*)-citramalate concentration in ECM-SpMAE1, ECM-pGK423, and ECM at 96 h. All data are presented as the average of three independent experiments. Error bars represent standard deviations.


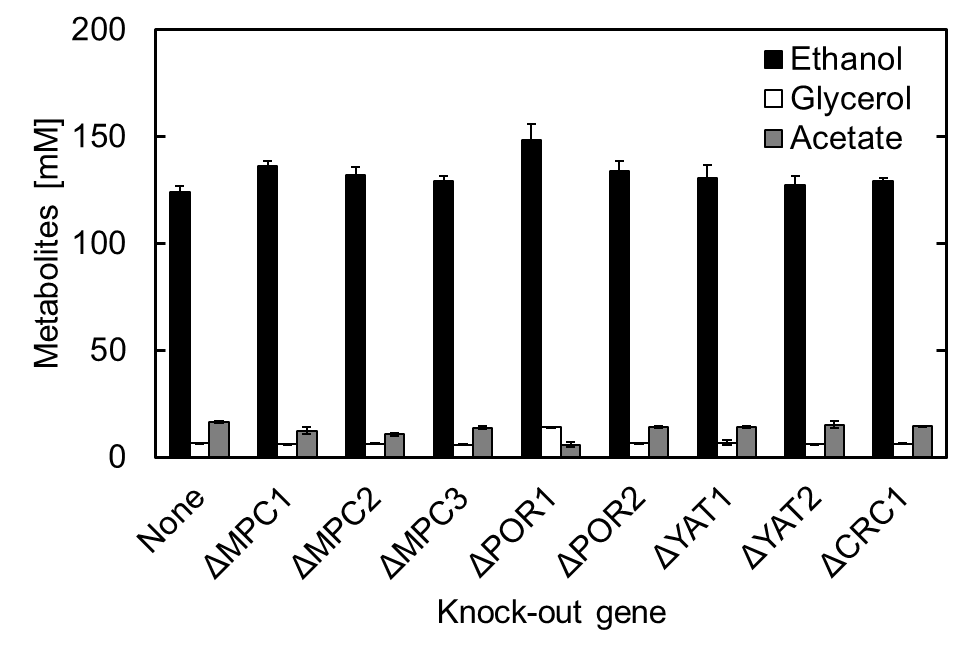


**Supplementary Fig. 2. Byproduct production by gene-knockout strains**

The production of ethanol, glycerol, and acetate by eight gene-knockout strains at 96 h. All data are presented as averages of three independent experiments. Error bars represent standard deviations.


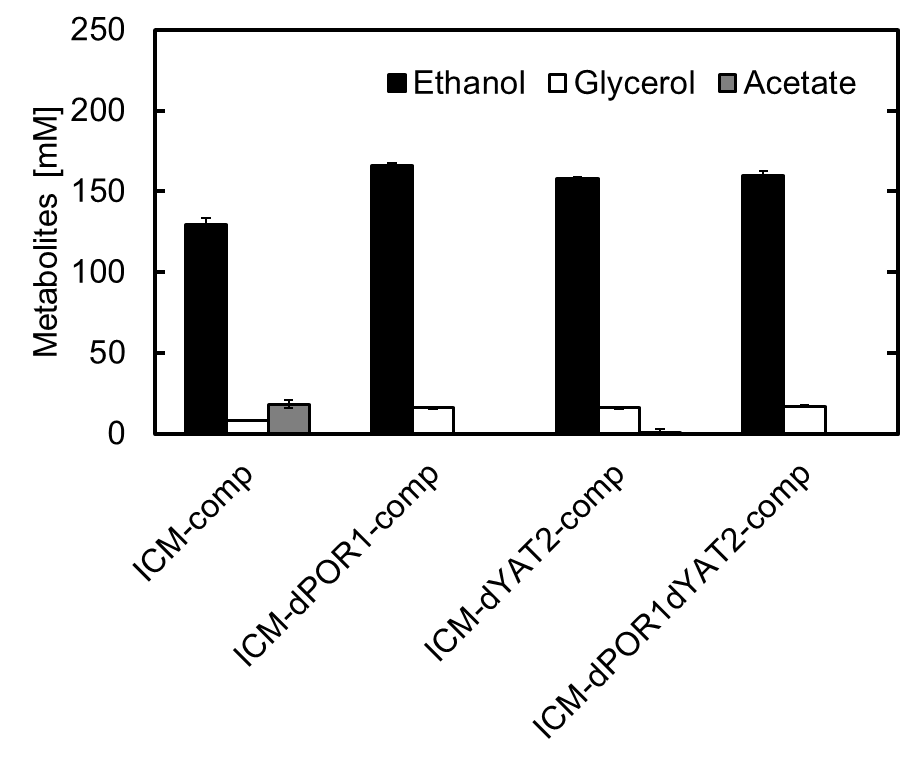


**Supplementary Fig. 3. Byproduct production by recombinant strains in test tube**

The production of ethanol, glycerol, and acetate at 96 h by ICM-comp, ICM-dPOR1-comp, ICM-dYAT2-comp, and ICM-dPOR1dYAT2-comp, respectively. All data are presented as the average of three independent experiments. Error bars represent standard deviations.
